# TargetRNA3: predicting prokaryotic RNA regulatory targets with machine learning

**DOI:** 10.1186/s13059-023-03117-2

**Published:** 2023-12-01

**Authors:** Brian Tjaden

**Affiliations:** Department of Computer Science, Wellesley College, Wellesley, MA USA

**Keywords:** sRNA, Regulation, Target prediction, Prokaryotes

## Abstract

Small regulatory RNAs pervade prokaryotes, with the best-studied family of these non-coding genes corresponding to *trans*-acting regulators that bind via base pairing to their message targets. Given the increasing frequency with which these genes are being identified, it is important that methods for illuminating their regulatory targets keep pace. Using a machine learning approach, we investigate thousands of interactions between small RNAs and their targets, and we interrogate more than a hundred features indicative of these interactions. We present a new method, TargetRNA3, for predicting targets of small RNA regulators and show that it outperforms existing approaches. TargetRNA3 is available at https://cs.wellesley.edu/~btjaden/TargetRNA3.

**Supplementary Information:**

The online version contains supplementary material available at 10.1186/s13059-023-03117-2.

## Background

Small regulatory RNAs (sRNAs) are widespread in prokaryotes [1]. In *Escherichia coli* and *Salmonella enterica* serovar Typhimurium, for example, several hundred sRNAs have been characterized, comparable to the number of transcription factors [2]. The vast majority of sRNAs act as post-transcriptional regulators by base pairing with target mRNAs, thereby modulating the translation or stability of the targets [3]. The products of a sRNA gene normally interact with multiple mRNAs, enabling sRNAs to effect broad cellular responses. Altogether, more than half of genes in a genome may be subject to sRNA-mediated regulation [4].

While sRNAs are important components of the regulatory landscape, their annotation can be challenging, in part owing to their diversity in size and function and extent of conservation. For example, in some organisms, the action of sRNAs is heavily dependent on sRNA-binding proteins, such as Hfq, ProQ, and CsrA [5], whereas in other organisms, it is unclear that sRNA-binding proteins play a significant role [6]. Among prokaryotes, sRNAs and their targets of regulation have been characterized much more extensively in bacteria than in archaea [7, 8]. While the number of identified sRNAs in prokaryotes has exploded in recent years, thanks in part to advances in RNA-seq strategies, a major challenge continues to be the effective elucidation of their functional roles and regulatory targets.

To help address this challenge, a number of experimental approaches have been developed for the purpose of large-scale target identification. MAPS (MS2 affinity purification coupled with RNA sequencing) fuses an MS2 tag to a sRNA followed by purification and sequencing to determine the targets of a sRNA [9]. RIL-seq (RNA interaction by ligation and sequencing) detects sRNA:target duplexes by co-immunoprecipitation with Hfq followed by ligation and sequencing [10]. GRIL-seq (global small noncoding RNA target identification by ligation and sequencing), similar to RIL-seq, uses ligation and sequencing to identify sRNA:target interactions, and it does not require RNA-binding proteins in order to capture interactions [11]. Likewise, CLASH (UV-crosslinking, ligation, and sequencing of hybrids) uses cross-linking followed by ligation and sequencing to capture sRNA:target interactions [12]. All of these approaches can be applied in vivo to identify sRNA:target interactions globally. Overall, while these high-throughput experimental methods can be applied genome-wide and they have substantially increased the number of validated sRNA:target interactions, they do not scale with the exploding number of identified sRNAs throughout prokaryotes. Thus, computational approaches, which are more efficient than experimental methods, can be a useful first step in helping characterize targets of *trans*-acting regulatory RNAs in prokaryotes.

There are a number of existing computational tools for predicting RNA-RNA interactions in different domains of life [13, 14] and in prokaryotes specifically [15]. For predicting targets of sRNA regulation throughout a prokaryotic genome, TargetRNA was the first such tool, utilizing the energy of hybridization between a sRNA and a target as well as a seed region of consecutive base pairs to identify regulatory interactions [16]. RNAup determines the thermodynamics of a sRNA and target interaction by combining their hybridization energy with the structural accessibility of the binding regions [17]. IntaRNA, which improves upon the execution time of the approach used by RNAup and also incorporates seed regions into its prediction calculations, is one of the more precise tools at estimating the interacting region and corresponding nucleotides that participate in hybridization between a sRNA and target [18]. CopraRNA is a leading tool that rigorously incorporates the conservation of sRNA:mRNA interactions across species to determine its predictions [19]. sTarPicker [20] and sRNARFTarget [21] both employ machine learning approaches to make predictions, with sTarPicker using an ensemble classifier based on the Tclass system [22] and sRNARFTarget using random forests as its machine learning foundation. SPOT uses an ensemble approach, combining several of the abovementioned tools to enhance predictive performance [23]. Each of these tools has its pros and cons. For instance, CopraRNA is one of the most accurate tools in identifying interactions; however, it is only able to make predictions for highly conserved sRNAs and targets, and it is prohibitively slow to run for large numbers of sRNAs. Altogether, existing tools share many of the same challenges. In general, these tools consider only a few features predictive of sRNA:target interactions, often focusing on the thermodynamics of hybridization between the two RNAs, and all have high false-positive rates. Additionally, existing approaches were designed and evaluated based on relatively small sets of sRNAs and interactions, so their effectiveness beyond a few model organisms is not well understood.

In this study, we gathered a large set of experimentally determined sRNA:target interactions, substantially larger than sets used to build and assess previous tools for predicting sRNA targets. We then investigated a variety of features that may be predictive of interactions. Using this rich set of data on sRNAs and their targets, we trained a machine learning model to distinguish interactions from non-interactions. We show that our approach, TargetRNA3, identifies targets of sRNA action more accurately than existing approaches. At the same false-positive rate as other tools, TargetRNA3 identifies significantly more true targets, and correspondingly, when identifying the same number of targets as other tools, TargetRNA3 has a significantly lower false-positive rate. TargetRNA3 can be applied to all sRNAs, regardless of whether they are conserved, and it is dramatically faster than other leading tools. TargetRNA3 can be used via a web interface at https://cs.wellesley.edu/~btjaden/TargetRNA3 [24].

## Results

### Features of target interactions

In order to evaluate features indicative of interactions between sRNAs and their regulatory targets, we compiled a set of 4386 sRNA:target interactions for which there is experimental evidence. The 4386 interactions come from 77 sRNAs in 13 different genomes from 4 phyla (Additional file 1: Table S1). For the 77 sRNAs, we also looked at possible targets in their corresponding genomes for which we did not find experimental evidence of interaction. There are 325,162 pairs of sRNAs and possible targets in the 13 genomes without evidence of interaction. We consider these 325,162 pairs as non-interactions. Of course, some of these pairs that we label as non-interacting may indeed be regulatory interactions for which we have not yet found evidence. Thus, the false-positive rates we ultimately report may be over-estimates. Nonetheless, since most sRNAs have regulatory interactions with only a small percentage of all possible targets from their genome, we hypothesize that the number of false-negative labels is relatively modest.

For each of these 329,548 pairs of sRNAs and possible targets, we calculated values for 111 features that may be predictive of sRNA:target interactions. Most of the features have been used in other studies to predict interactions, though a few are new to this study. For instance, 64 of the features correspond to trinucleotide frequency differences as used by sRNARFTarget, and 17 of the features correspond to properties of the IntaRNA-predicted hybridization as suggested by sInterBase [25]. The complete set of 329,549 pairs of sRNAs and possible targets together with each of their 111 feature values is available in Additional file 1: Table S2, and details on the features are provided in Additional file 1: Table S3.

**Fig. 1:**
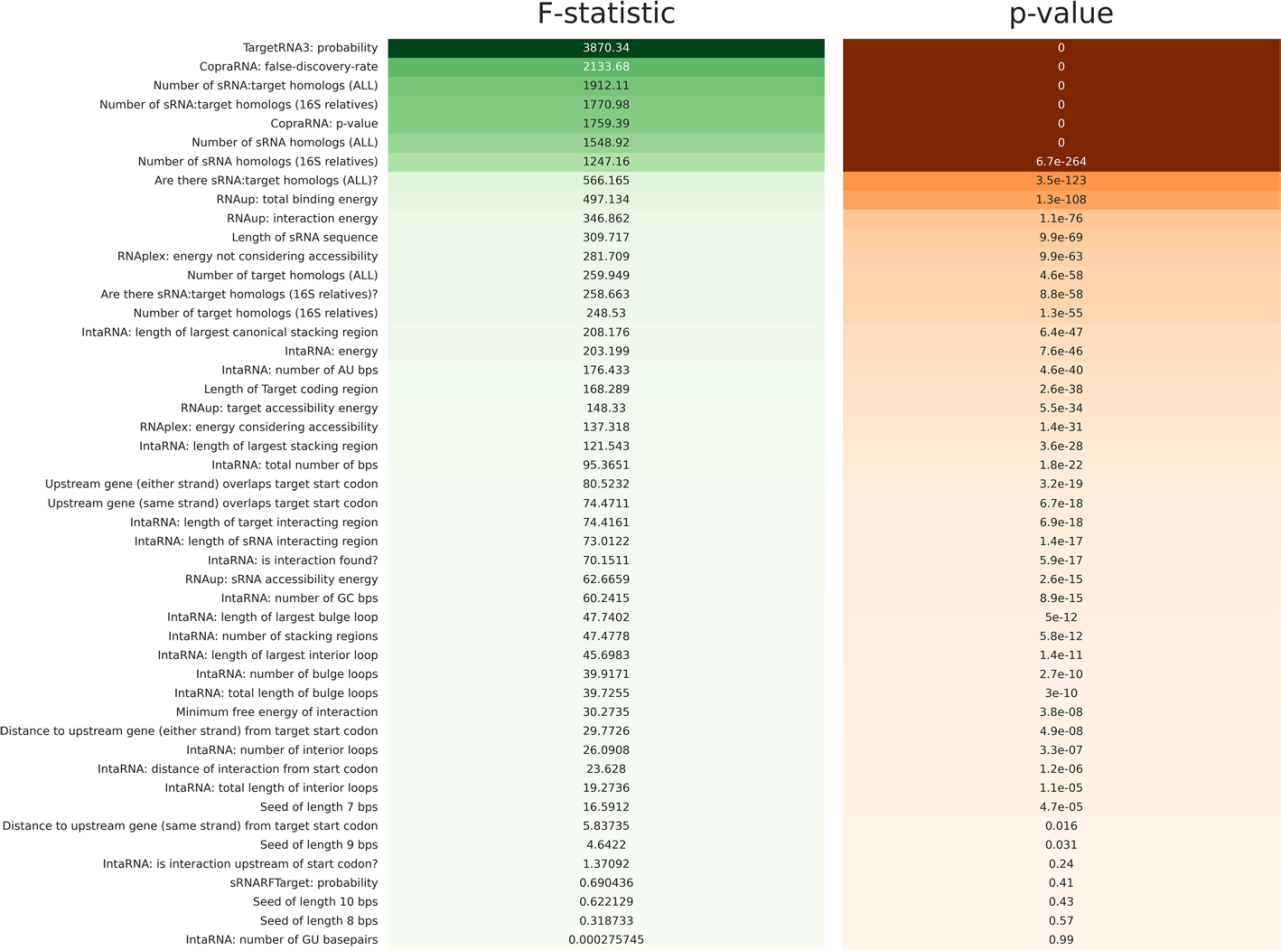
Relationship of features to evinced interactions. The *F*-statistic and corresponding *p*-value, as calculated using analysis of variance, are shown for each feature except for the 64 trinucleotide frequency differences. Higher *F*-statistics and lower *p*-values (more darkly shaded regions in the figure) indicate how well the feature discriminates interactions from non-interactions. For comparison, the first row shows the *F*-statistic and *p*-value for the probabilities reported by TargetRNA3

We then investigated combinations and subsets of the 111 features as well as the relationship of each feature with interactions and non-interactions (Additional file 1). For each feature, we used analysis of variance (ANOVA) to calculate its *F*-statistic and corresponding *p*-value demonstrating the feature’s relationship to whether interactions are evinced or not (Fig. 1) [26]. As Fig. 1 illustrates, some features are not informative in distinguishing interactions from non-interactions. For example, the existence of seed regions of length 8, 9, or 10 base pairs, which are used in several existing prediction tools and which correspond to consecutive base pairs in the sRNA and in the possible target that are perfectly complementary, does not contain substantial predictive power (*p*-values of 0.57, 0.031, and 0.43, respectively). In contrast, features related to homology appear to be important. Features capturing the conservation of a sRNA and its possible target have significant *p*-values as do the two features from CopraRNA, a tool which makes heavy use of homology in computing its *p*-value and false discovery rate. Similarly, features relating to the binding energy of a sRNA and possible target tend to be significant.

Based on the significance of different features in distinguishing interactions from non-interactions (Fig. 1) and the efficiency of calculating different features (Additional file 1: Table S3), we selected a subset of nine features that capture the key aspects of separating interactions from non-interactions and that can be computed rapidly. The nine features are shown in Additional file 1: Fig. S3 with their relationship to whether interactions are evinced or not based on ANOVA (Additional file 1: Fig. S3A) and based on correlation coefficient (Additional file 1: Fig. S3B).

### Machine learning algorithms

**Fig. 2:**
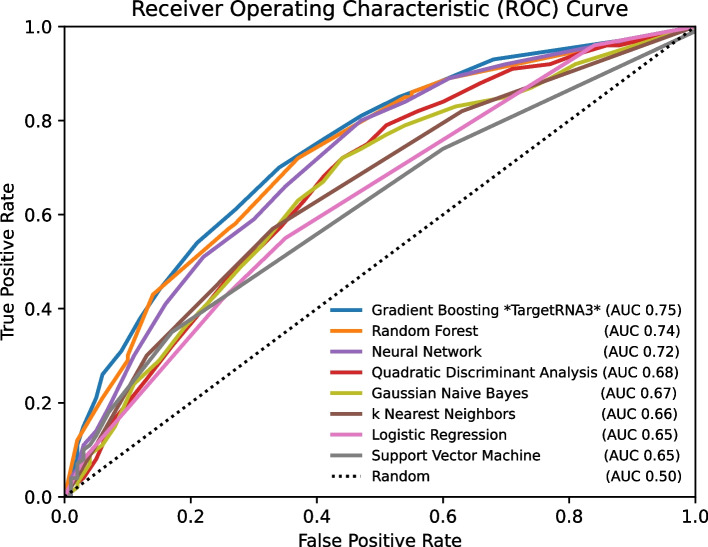
ROC curves showing the performance of different machine learning algorithms. The performance of 8 machine learning algorithms is illustrated by ROC curves. The abscissa axis corresponds to the false-positive rate, i.e., 1.0 − specificity. The ordinate axis corresponds to the true positive rate, i.e., the recall or sensitivity. Different thresholds for the values reported by an algorithm represent different points along the algorithm’s curve in the figure. The dotted line with unit slope indicates the performance of a naïve random algorithm. For each algorithm, the area under the curve (AUC) is indicated

Using our set of 329,548 pairs of sRNAs and possible targets, we explored 8 different machine learning algorithms and evaluated each algorithm for its ability to accurately identify sRNA:target interactions. Once trained, each algorithm reports a probability that any sRNA and possible target genuinely interact. Figure 2 shows the receiver operating characteristic (ROC) curves for the eight machine learning algorithms, indicating the trade-off between sensitivity (i.e., true-positive rate) and specificity (i.e., 1.0 − false-positive rate) at different probability thresholds, and Additional file 1: Table S4 provides additional statistics, including area under the ROC curve, F1 score, and Matthews correlation coefficient, indicating each algorithm’s performance. Based on these results (Fig. 2 and Additional file 1: Table S4), we found that the gradient boosting algorithm was one of the best performing at any threshold and, particularly, at probability thresholds corresponding to very low false-positive rates such as false-positive rates of 0.05 (the left-most region of Fig. 2). Given its robustness at different thresholds, its performance at low false-positive rates that are most relevant to target prediction, and its speed, we selected the gradient boosting algorithm for more careful investigation and as the basis for TargetRNA3.

**Fig. 3:**
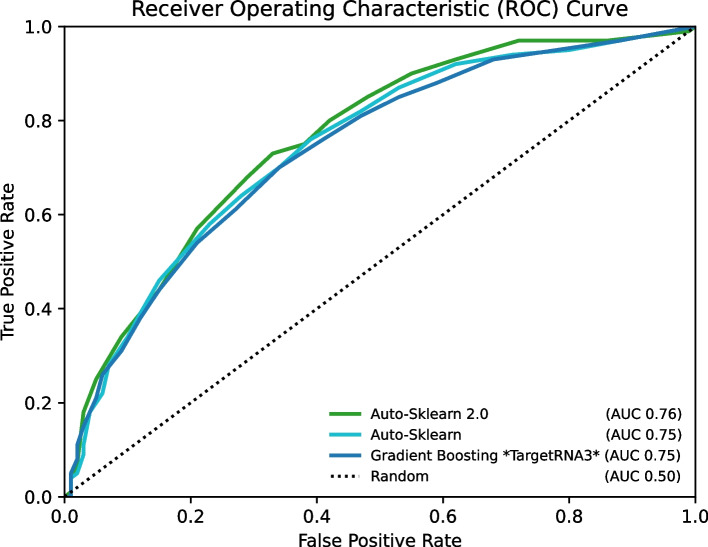
ROC curves comparing the performance of TargetRNA3 with AutoML. The performance of TargetRNA3 and two AutoML systems, Auto-Sklearn and Auto-Sklearn version 2.0, is illustrated by ROC curves. The abscissa axis corresponds to the false-positive rate, i.e., 1.0 − specificity. The ordinate axis corresponds to the true-positive rate, i.e., the recall or sensitivity. Different thresholds for the values reported by an algorithm represent different points along the algorithm’s curve in the figure. The dotted line with unit slope indicates the performance of a naïve random algorithm. For each algorithm, the area under the curve (AUC) is indicated

After identifying gradient boosting as the best of the eight algorithms that we considered for target prediction, we examined how its performance compared to that of an automated machine learning (AutoML) system, namely auto-sklearn [27]. auto-sklearn is a popular AutoML system that uses meta-learning and Bayesian optimization to determine the optimal learning algorithms and their associated hyperparameter optimizations in a combined search space. Thus, in contrast to a single machine learning algorithm such as gradient boosting, auto-sklearn explores a large set of algorithms and not just individually but in combinations as part of ensembles while simultaneously optimizing their parameters. Figure 3 shows the ROC curves for gradient boosting, which is used by TargetRNA3, and for both auto-sklearn [27] and auto-sklearn 2.0 [28]. While the AutoML approaches perform better than gradient boosting at most probability thresholds, their performance is comparable to gradient boosting at thresholds corresponding to very low false-positive rates, which are our foci when predicting sRNA:target interactions.

**Fig. 4:**
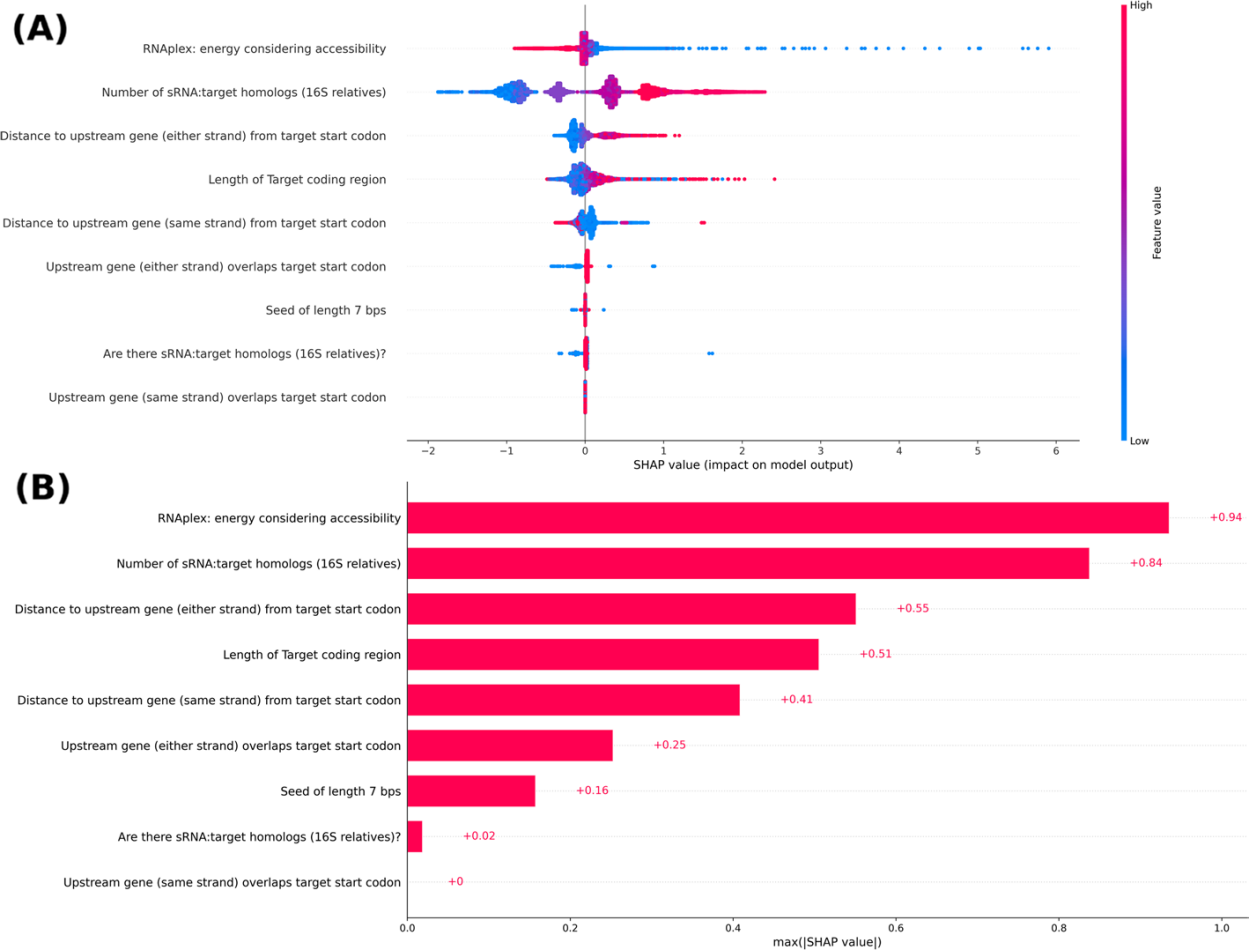
Contributions of features used by TargetRNA3. The results of SHAP analyses are shown indicating the contributions of features used by TargetRNA3 when making predictions. **A** For each of the nine features, the feature’s impact on the machine learning model’s output is shown by the distribution of the feature’s Shapley values. **B** For each of the nine features, the maximum absolute Shapley value over all interactions is indicated

Having examined different machine learning algorithms and their performance, we wanted to better understand the relative contribution of each feature toward distinguishing interactions, so we performed a SHAP (SHapley Additive exPlanations) analysis, which enables global measures of feature importance for a machine learning model [29]. Figure 4 illustrates the impact of different features on TargetRNA3’s predictions based on Shapley values. As indicated in Fig. 4, some features such as the energy of hybridization of the two interacting RNAs as determined by RNAplex (blue values in Fig. 4A for this feature correspond to large negative energies) and the number of sRNA:target homologs (red values in Fig. 4A for this feature correspond to large numbers of homologs) contribute more toward TargetRNA3’s predictions and some features such as whether the stop codon of a target’s upstream gene overlaps the target’s start codon contribute little toward TargetRNA3’s predictions.

### Comparison with other target prediction methods

**Fig. 5:**
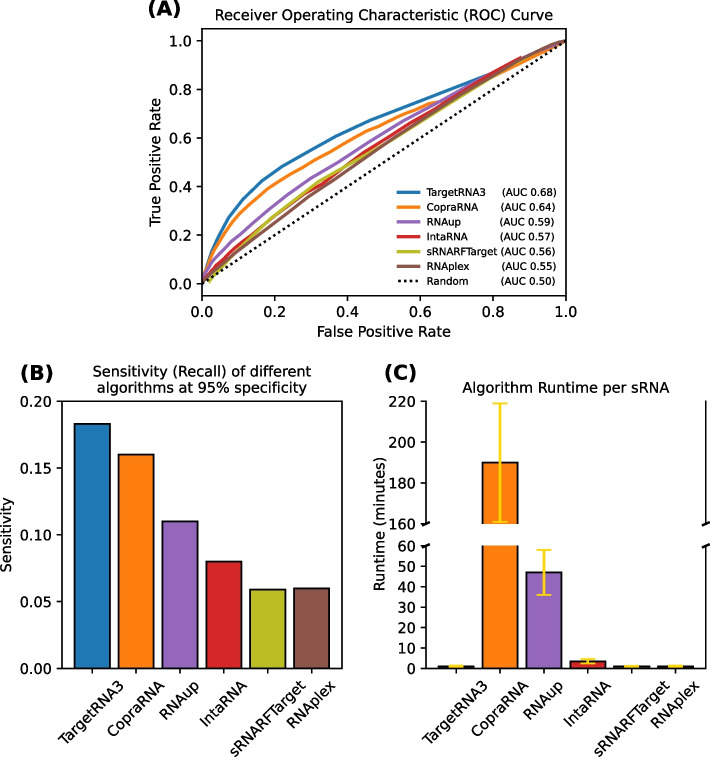
Performance comparison of TargetRNA3 and existing tools for predicting targets of sRNA regulation. The performance of TargetRNA3 and five existing tools (CopraRNA, RNAup, IntaRNA, SRNARFTarget, and RNAplex) when predicting sRNA targets is shown. **A** ROC curves for the six tools are illustrated. The abscissa axis corresponds to the false-positive rate, i.e., 1.0 − specificity. The ordinate axis corresponds to the true-positive rate, i.e., the recall or sensitivity. Different thresholds for the values reported by a tool represent different points along the tool’s curve in the figure. The dotted line with unit slope indicates the performance of a naïve random tool. **B** A particular point along each curve in **A**, specifically the point at which each of the six curves intersects the vertical line corresponding to a false-positive rate of 0.05. **B** The sensitivity, i.e., recall or true-positive rate, is shown for the six tools when their specificity is 95%, i.e., their false-positive rate is 0.05. **C** The mean runtime in minutes per sRNA is shown for the six tools, with yellow error bars corresponding to the standard error

To assess how TargetRNA3 compares to other approaches for predicting sRNA:target interactions, we interrogated the performance of TargetRNA3, CopraRNA [19], RNAup [17], IntaRNA [18], RNAplex [30], and sRNARFTarget [21]. It is worth noting that CopraRNA has shown some of the best performance in the past at identifying target interactions [15], and sRNARFTarget is a recent approach for target prediction that also employs machine learning and uses a set of features unique among the tools—namely the difference in frequency for each of the 64 trinucleotides between the sRNA sequence and a possible target sequence. Detailed scores reported by each of these 6 algorithms on all 329,548 pairs in our dataset are reported in Additional file 1: Table S2. Figure 5A illustrates the ROC curves for each of the six algorithms as well as the area under the curve (AUC) for each. While ROC curves show true-positive rate and false-positive rate performance at different thresholds, we are particularly interested in low false-positive rates, so we considered the true-positive rate (i.e., sensitivity) of each of the six algorithms at a specific false positive rate of 0.05 (Fig. 5B). We also probed the runtime, per sRNA, of each of the six algorithms (Fig. 5C). As shown in Fig. 5 and Additional file 1: Table S5, TargetRNA3 had the best performance overall and critically at low false-positive rates. TargetRNA3 has the added benefit of one of the fastest runtimes, which is not accidental, since we selected 9 features out of 111 for TargetRNA3 where runtime of computing a feature was one of the considerations in selecting it.

## Discussion

Most existing tools for computational prediction of targets of sRNA regulation in prokaryotes were created and tested based on a relatively small number of sRNA:target interactions, often around 100 such interactions or fewer. With the recent application of experimental approaches toward global detection of sRNA:target interactions, such as RIL-seq, MAPS, and CLASH, a more extensive array of interactions has been cataloged. Here, we gather a dataset with more than 4000 experimentally determined interactions and more than 300,000 putative non-interactions. For each of these interactions, we computed values for more than a hundred different features that may be predictive of sRNA:target regulatory interactions. We hope this rich dataset will be a resource to the community for helping improve biocomputational examination of sRNA regulatory interactions.

As part of our investigations, we interrogated the features in the dataset, and we found that some features commonly used for target prediction, such as longer seed lengths, did not have substantial predictive power in discerning sRNA:target interactions. In contrast, other features, such as the conservation of the sRNA and target and thermodynamics of their interaction, had highly significant predictive power. Thus, we were able to identify a subset of features that together are effective at distinguishing interactions from non-interactions. Using this subset of features with our large dataset of interactions, we explored eight popular machine learning algorithms to understand how well interactions could be predicted. We also explored a more advanced autoML approach that creates an ensemble of different machine learning algorithms while simultaneously optimizing their parameters. Ultimately, we found predictive performance was relatively robust with respect to the choice of machine learning algorithm. Thus, we chose one of the best performing and most efficient algorithms, gradient boosting, and we used this as the foundation for our new method, TargetRNA3, for predicting targets of sRNA regulation.

After training TargetRNA3’s machine learning model on the large dataset of interactions, we assessed TargetRNA3’s ability to accurately predict sRNA targets by comparing TargetRNA3’s performance with that of other leading tools. TargetRNA3 outperformed the existing tools, consistently achieving lower false-positive rates. Additionally, while some existing tools have limitations such as only working with sRNAs for which multiple homologs can be identified or requiring hours to execute on a single sRNA thereby constraining searches with large numbers of sRNAs, TargetRNA3 does not have these limitations.

One of the challenges with our approach, however, is that the large dataset on which our machine learning model is trained contains many interactions that were observed through global methods, such as RIL-seq, CLASH, and GRIL-seq, which capture sRNA interactions with other RNAs but not necessarily functional regulation. Some studies have reported good but not perfect relationships between the interactions identified by these global methods and regulatory effects [10, 12, 31]. Thus, it is important to note that some predictions from our model may correspond to interactions that are non-functional.

TargetRNA3 has other limitations, though these are not necessarily unique to TargetRNA3 but common among sRNA target prediction tools. For example, while TargetRNA3’s false-positive rate is lower than other tools, it is still substantial. As increasing numbers of interactions are characterized experimentally, our ability to distinguish interactions from non-interactions will improve and false-positive rates for computational target prediction should decrease. But, presently, computational approaches such as TargetRNA3 continue to suffer from significant false-positive rates. Also, it is difficult to assess how effective TargetRNA3 and other tools are at predicting interactions throughout the range of disparate prokaryotes. Most existing tools were created and assessed on data from one or two genomes. TargetRNA3 is trained on data from 13 genomes. Such few genomes are not representative of the diversity across prokaryotes. Further, the 4000+ interactions used to construct TargetRNA3 may be enriched for sRNAs that rely on ribosome-binding proteins such as Hfq and ProQ or may be enriched for highly conserved sRNAs or may have other biases that are not indicative of sRNA action across the range of prokaryotes. In archaea in particular, relatively few interactions have been characterized experimentally, mostly by differential expression with sRNA deletion mutants or pulse expression rather than by capturing direct interactions such as with RIL-seq [32–34]. Thus, it is beneficial for computational tools to use increasingly large and diverse sets of interactions to improve their precision and broaden their applicability.

## Conclusions

Computational approaches for characterizing biological phenomena such as RNA interactions are generally more accurate when designed and evaluated on large and diverse sets of data. Thus, to help advance the state of computational prediction of sRNA regulatory targets in prokaryotes, we gathered a large set of sRNA:target interactions and computed values for a constellation of features indicative of regulatory interactions. Using this rich dataset, we trained a machine learning model to distinguish interactions from non-interactions. Based on this machine learning model, we built a tool, TargetRNA3, to predict targets of sRNA regulation. We found that TargetRNA3 consistently outperforms the existing tools. To enable ease of use and broad applicability, we designed a user-friendly web interface for executing TargetRNA3 that normally returns results in a matter of seconds. We hope that our machine learning analysis based on a large dataset of interactions will be a useful resource to the community of scientists studying RNA regulation in prokaryotes.

## Methods

TargetRNA3 is implemented in Python and makes heavy use of the scikit-learn [35] library to execute supervised classification machine learning algorithms. To generate our dataset of interactions (Additional file 1: Table S2), we combined data from sInterBase [25], sRNARFTarget [21], and sRNATarBase 3.0 [36] and retained only those interactions for which there was experimental evidence and for which the sRNA had multiple targets. The dataset was split into training data corresponding to 65 sRNAs and testing data corresponding to 12 sRNAs such that none of the genomes for the 12 testing sRNAs were represented in the training data. The training data were further split using five-fold cross-validation into training data and validation data, where training data were used to train the machine learning models and validation data were used to tune hyperparameters and evaluate different machine learning algorithms. Once the gradient boosting algorithm was selected and hyperparameters were tuned, testing data were used to assess TargetRNA3’s performance in comparison with existing tools for predicting targets of sRNA regulation.

Since the dataset contains highly imbalanced classes, i.e., 4385 (1%) interactions and 325,162 (99%) non-interactions, we experimented with mitigating the imbalance using random undersampling and the synthetic minority over-sampling technique (SMOTE) [37, 38]. We observed the best performance when randomly undersampling the non-interactions where the class imbalance is 25% interactions and 75% non-interactions, and consequently, we used this distribution of data going forward. When initially comparing eight different supervised classification algorithms from sklearn [35], namely gradient boosting, logistic regression, random forests, neural networks, *k*-nearest neighbors, support vector machines, Gaussian-naïve Bayes, and quadratic discriminant analysis, we used default parameter settings. After selecting gradient boosting as the best performing of these algorithms, based on tuning hyperparameters, we required a minimum of five samples per leaf node rather than the default value of 1 sample, which helps alleviate overfitting. When comparing to auto-sklearn [28], we used default parameter settings except that we increased the memory available for auto-sklearn from 3 to 12 Gb. Further, since auto-sklearn runs for a user-specified length of time while constantly exploring the search space of machine learning algorithms and their optimal hyperparameter values, we ran auto-sklearn for 300s on each execution.

For comparative genomics analyses, we use all prokaryotic genomes in RefSeq labeled as “reference genomes” or “representative genomes,” of which there are 4265 such bacterial genomes and 279 such archaeal genomes [39]. When executing CopraRNA version 2.1.2 [19], RNAup version 2.6.1 [30], IntaRNA version 2.3.1 [40], and RNAplex version 2.5.0 [41], we used default parameter settings and searched for target interactions from 200 nucleotides upstream to 100 nucleotides downstream of the start of the target. When executing sRNARFTarget [21], we used default parameter settings and searched for targets using the complete target sequence, consistent with how the sRNARFTarget system was designed [21]. When searching for homologs, we used BLAST + version 2.13.0 [42] and only retained hits with an *E*-value less than or equal to 0.01.

As input, TargetRNA3 requires a genome with gene annotations and a sRNA sequence. As output, TargetRNA3 produces a ranked list of targets, including a predicted structure and energy of the sRNA:target interaction, a probability as determined from the machine learning model that there is a regulatory interaction between the sRNA and target, and a corresponding *p*-value. TargetRNA3’s *p*-values are determined from the cumulative distribution function of a log-normal distribution fit to the interaction probabilities. TargetRNA3 also outputs plots indicating regions of the sRNA that participate in target interactions and regions of the sRNA that are conserved in other genomes.

### Supplementary Information

**Additional file 1:** Supplementary material. Principal component analysis. **Fig. S1.** Variance in data explained by principal components. **Fig. S2.** Interaction data with respect to most significant principal components. **Fig. S3.** Relationships to evinced interactions of select features used by TargetRNA3. **Table S1.** The table shows the phylum and class of the 13 genomes investigated in this study and the number of sRNAs from each genome. **Table S2.** The table, consisting of a large dataset (a CSV file approximately 340 MBs in size) with information on 329,548 possible sRNA:target interactions, is available at https://doi.org/10.7910/DVN/2Q8YRF. **Table S3.** The table provides a more detailed description of the 118 columns from the large dataset found in Table S2. The first five columns in Table S2 identify each sRNA and candidate target. The following 111 columns in Table S2 correspond to features that may be used for predicting whether a sRNA interacts with a candidate target. The penultimate column in Table S2 contains the probability that a sRNA and candidate target interact, as predicted by TargetRNA3. The final column in Table S2 indicates whether or not there is experimental evidence that a sRNA and candidate target interact. Features in the table below whose values were computed using an existing computational tool are indicated. The runtime to calculate each feature on a genome-wide scale is described in the table below as fast (generally requiring seconds or less), medium (generally requiring minutes), or slow (generally requiring one or more hours). **Table S4.** The table indicates the performance of 8 different machine learning algorithms using different measures: area under the ROC curve (AUC), F1 score (F1), Matthews correlation coefficient (MCC), sensitivity at a false positive rate of 5%, and the time in seconds required for training the machine learning model. **Table S5.** The table indicates the performance of 6 different tools for predicting targets of sRNA regulation.

**Additional file 2.** Peer review history.

## Data Availability

Data, software, and documentation are available under the MIT license at GitHub https://github.com/btjaden/TargetRNA3 [43]. The DOI for the source version used in this article is https://doi.org/10.5281/zenodo.8075839 [44].
