# Supplementary material for: TargetRNA3: predicting prokaryotic RNA regulatory targets with machine learning
Source: Genome Biol. 2023 Dec 1;24:276. doi: 10.1186/s13059-023-03117-2 (PMC10691042; doi:10.1186/s13059-023-03117-2)
Supplement: Supplementary file 1 — Additional file 1: Supplementary material. Principal component analysis. Fig. S1. Variance in data explained by principal components. Fig. S2. Interaction data with respect to most significant principal components. Fig. S3. Relationships to evinced interactions of select features used by TargetRNA3. Table S1. The table shows the phylum and class of the 13 genomes investigated in this study and the number of sRNAs from each genome. Table S2. The table, consisting of a large dataset (a CSV file approximately 340 MBs in size) with information on 329,548 possible sRNA:target interactions, is available at https://doi.org/10.7910/DVN/2Q8YRF. Table S3. The table provides a more detailed description of the 118 columns from the large dataset found in Table S2. The first five columns in Table S2 identify each sRNA and candidate target. The following 111 columns in Table S2 correspond to features that may be used for predicting whether a sRNA interacts with a candidate target. The penultimate column in Table S2 contains the probability that a sRNA and candidate target interact, as predicted by TargetRNA3. The final column in Table S2 indicates whether or not there is experimental evidence that a sRNA and candidate target interact. Features in the table below whose values were computed using an existing computational tool are indicated. The runtime to calculate each feature on a genome-wide scale is described in the table below as fast (generally requiring seconds or less), medium (generally requiring minutes), or slow (generally requiring one or more hours). Table S4. The table indicates the performance of 8 different machine learning algorithms using different measures: area under the ROC curve (AUC), F1 score (F1), Matthews correlation coefficient (MCC), sensitivity at a false positive rate of 5%, and the time in seconds required for training the machine learning model. Table S5. The table indicates the performance of 6 different tools for predicting targets of sRNA regulati [file 13059_2023_3117_MOESM1_ESM.pdf]

Supplementary Material

TargetRNA3: Predicting prokaryotic RNA regulatory targets with machine learning

Contents

- Principal component analysis
- Fig. S1. Variance in data explained by principal components
- Fig. S2. Interaction data with respect to most significant principal components
- Fig. S3. Relationships to evinced interactions of select features used by TargetRNA3
- Table S1
- Table S2
- Table S3
- Table S4
- Table S5

Principal Component Analysis

To better understand the relationships between the 111 features, we performed a principal component analysis (PCA). Instead of representing points (sRNA and candidate target pairs) by their 111 feature values, we consider points with respect to 111 principal components, i.e., the eigenspace determined from the covariance matrix. We then remove principal components in order of increasing corresponding eigenvalues. Fig. S1 shows the percent of variance explained when different numbers of principal components (from 1 to 111) are used as the dimensionality of the points is decreased. The figure shows that the features are not independent of each other, for instance more than half of the variance in the data can be explained by fewer than 20 of the 111 principal components. This is unsurprising since so many features are related, e.g., a number of features capture some form of the strength of hybridization between the two RNA sequences. Fig. S2 shows the data points (sRNA and candidate target pairs) projected into a space defined by the two or three most significant principal components. Visually, there does not appear to be much separation between points corresponding to interactions and points corresponding to non-interactions in these low dimensional spaces. Ultimately, we did not further pursue the use of PCA because it requires calculating all 111 features, and some of the features are too computationally slow to calculate in real time for our purposes.

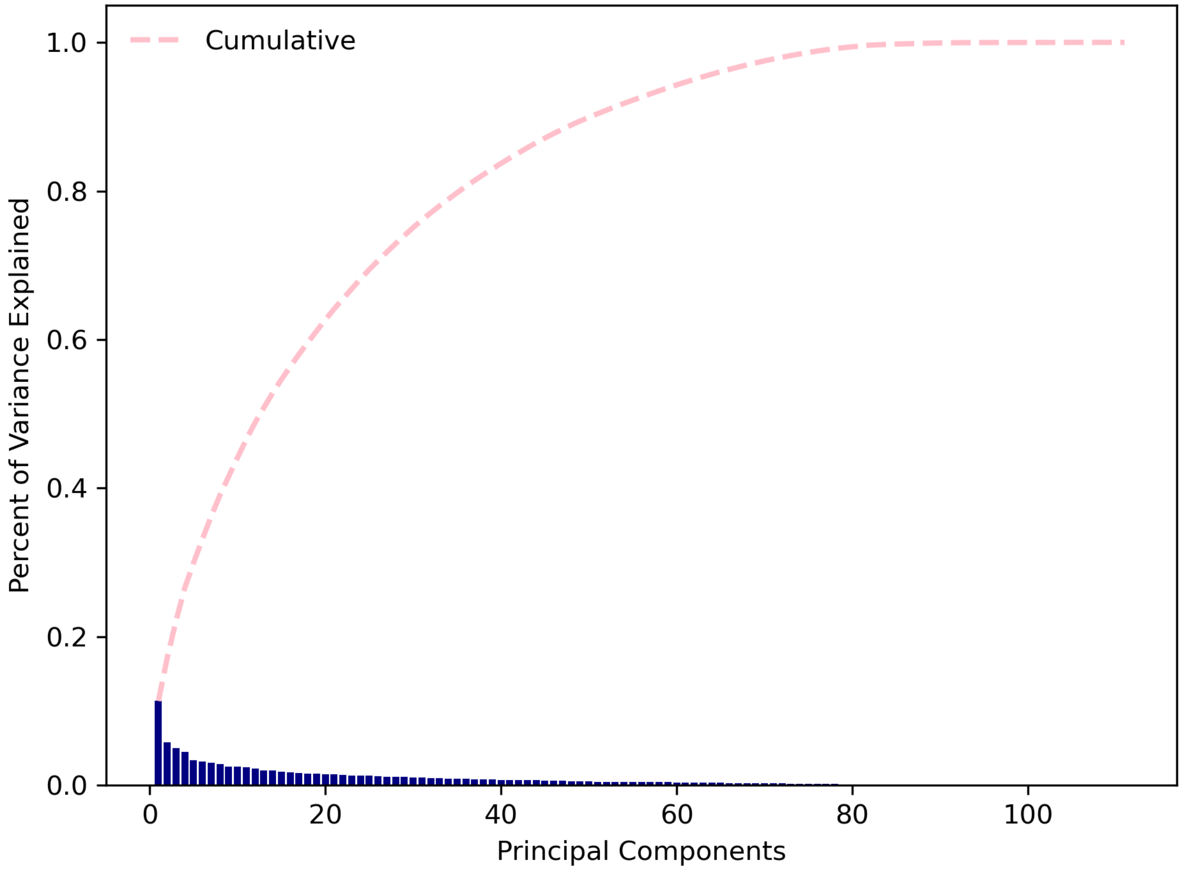

Fig. S1. Variance in data explained by principal components.

A principal component analysis was performed on the 111-dimensional data. The percent of variance explained by different principal components in decreasing significance (from left to right) is shown in the figure. The dashed line shows the cumulative percentage of variance explained by increasing numbers of principal components.

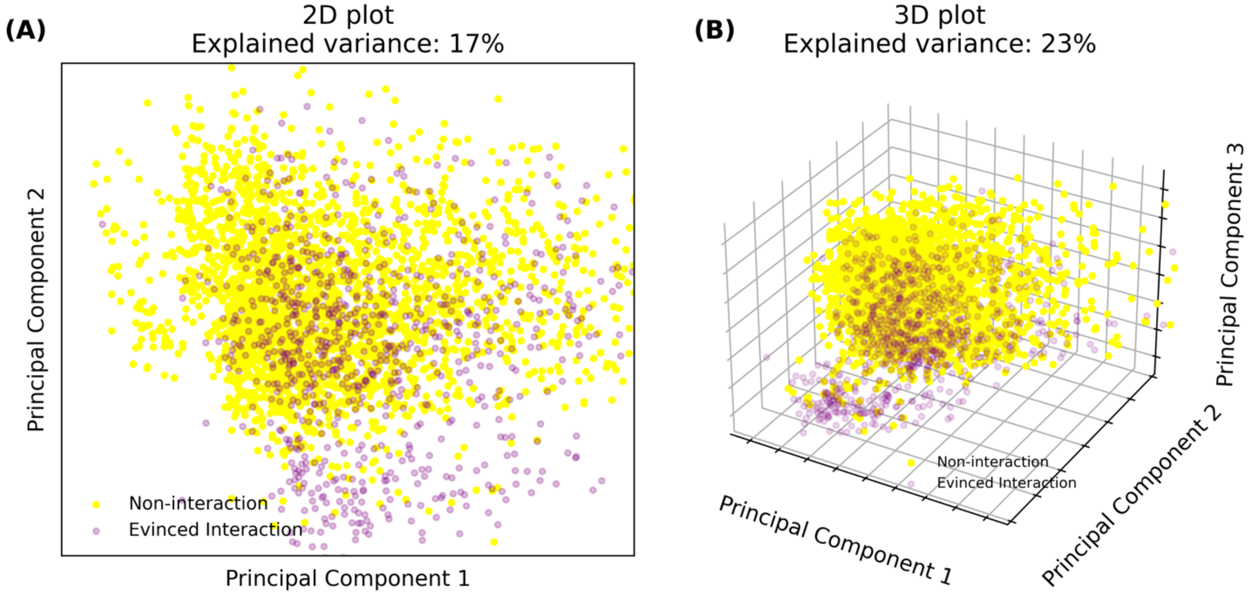

Fig. S2. Interaction data with respect to most significant principal components.

Following principal component analysis on the 111-dimensional data, possible sRNA:target interactions are plotted with respect to the two (A) or three (B) most significant principal components. Yellow points correspond to non-interactions and purple points correspond to interactions.

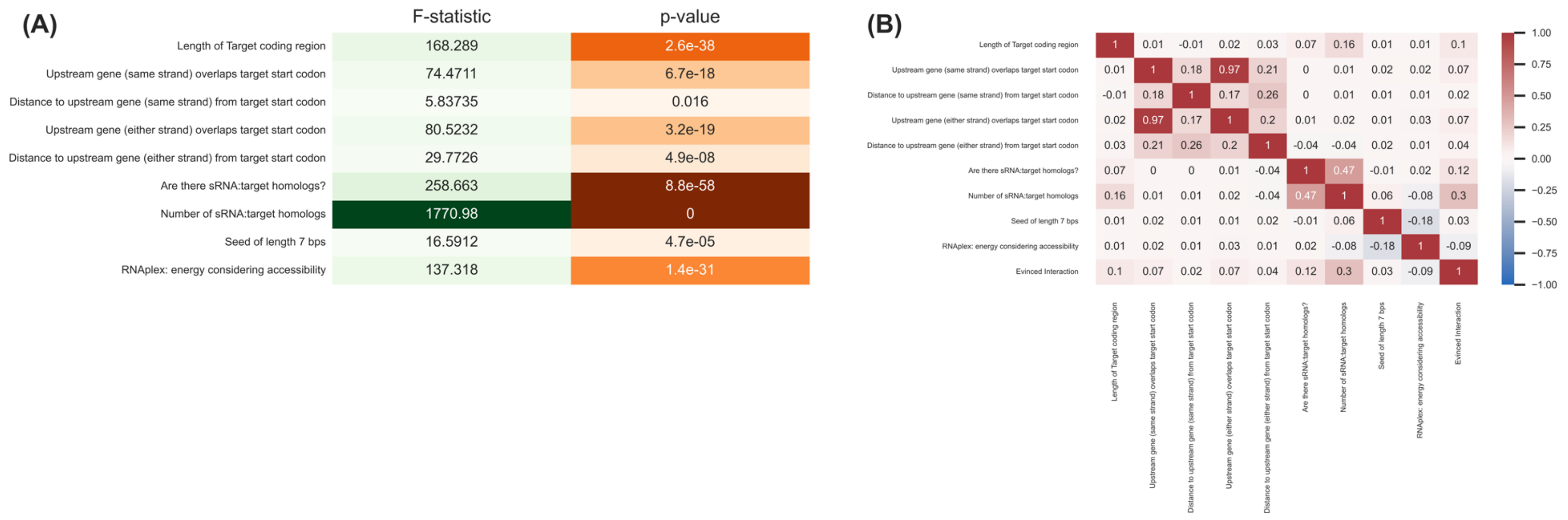

Fig. S3. Relationships to evinced interactions of select features used by TargetRNA3.

(A) The F-statistic and corresponding *p*-value, as calculated using analysis of variance, are shown for the 9 features used by TargetRNA3. Higher F-statistics and lower *p*-values (more darkly shaded regions in the figure) indicate how well the feature discriminates interactions from non-interactions. (B) The Pearson correlation coefficient is shown indicating the correlation of each of the 9 features used by TargetRNA3 with each of the other features used by TargetRNA3. The final row and column correspond to the correlation of each feature with whether interactions are evinced or not.

Table S1.

The table shows the phylum and class of the 13 genomes investigated in this study and the number of sRNAs from each genome.

Phylum	Class	Genome	Accession	Number of sRNAs
Pseudomonadota	Alphaproteobacteria	Agrobacterium fabrum str. C58	GCF_000092025.1	1
Pseudomonadota	Gammaproteobacteria	Azotobacter vinelandii DJ	GCF_000021045.1	1
Bacillota	Bacilli	Bacillus subtilis subsp. subtilis str. 168	GCF_000009045.1	1
Pseudomonadota	Gammaproteobacteria	Escherichia coli str. K-12 substr. MG1655	GCF_000005845.2	61
Actinomycetota	Actinomycetes	Mycobacterium tuberculosis H37Rv	GCF_000195955.2	1
Pseudomonadota	Gammaproteobacteria	Pasteurella multocida	GCF_000006825.1	1
Pseudomonadota	Gammaproteobacteria	Pseudomonas aeruginosa PAO1	GCF_000006765.1	2
Pseudomonadota	Gammaproteobacteria	Salmonella enterica subsp. enterica serovar Typhimurium str. 14028S	GCF_000022165.1	1
Pseudomonadota	Gammaproteobacteria	Salmonella enterica subsp. enterica serovar Typhimurium str. LT2	GCF_000006945.2	1
Pseudomonadota	Gammaproteobacteria	Salmonella enterica subsp. enterica serovar Typhimurium str. SL1344	GCF_000210855.2	2
Bacillota	Bacilli	Staphylococcus aureus subsp. aureus 6850	GCF_000462955.1	1
Cyanobacteriota	Cyanophyceae	Synechocystis PCC 6803	GCF_000009725.1	2
Pseudomonadota	Gammaproteobacteria	Vibrio cholerae O1 biovar El Tor str. N16961	GCF_000006745.1	2

Table S2.

The table, consisting of a large dataset (a CSV file approximately 340 MBs in size) with information on 329,548 possible sRNA:target interactions, is available at

https://doi.org/10.7910/DVN/2Q8YRF

Table S3.

The table provides a more detailed description of the 118 columns from the large dataset found in Table S2. The first five columns in Table S2 identify each sRNA and candidate target. The following 111 columns in Table S2 correspond to features that may be used for predicting whether a sRNA interacts with a candidate target. The penultimate column in Table S2 contains the probability that a sRNA and candidate target interact, as predicted by TargetRNA3. The final column in Table S2 indicates whether or not there is experimental evidence that a sRNA and candidate target interact. Features in the table below whose values were computed using an existing computational tool are indicated. The runtime to calculate each feature on a genome-wide scale is described in the table below as fast (generally requiring seconds or less), medium (generally requiring minutes), or slow (generally requiring one or more hours).

#	Column	Is Feature?	Computational Tool	Time to Compute	Description
1	Genome				Name of genome
2	sRNA Accession				Genomic accession of replicon where sRNA gene resides
3	sRNA				Name of sRNA
4	Target Accession				Genomic accession of replicon where target gene resides
5	Target				Name of target
6	Length of sRNA sequence	Y		Fast	Length of the sRNA sequence in nucleotides
7	Length of Target coding region	Y		Fast	Length of the target sequence in nucleotides
8	Upstream gene (same strand) overlaps target start codon	Y		Fast	Whether (1) or not (0) the upstream gene on the same strand overlaps the start of the target
9	Distance to upstream gene (same strand) from target start codon	Y		Fast	Distance in nucleotides from the start of the target to the upstream gene on the same strand
10	Upstream gene (either strand) overlaps target start codon	Y		Fast	Whether (1) or not (0) the upstream gene on the either strand overlaps the start of the target
11	Distance to upstream gene (either strand) from target start codon	Y		Fast	Distance in nucleotides from the start of the target to the upstream gene on the either strand
12	Number of sRNA homologs (ALL)	Y		Medium	Number of sRNA homologs (max 100) when searching all reference and representative genomes in RefSeq
13	Number of target homologs (ALL)	Y		Medium	Number of target homologs (max 100) when searching all reference and representative genomes in RefSeq
14	Are there sRNA:target homologs (ALL)?	Y		Medium	Are there any sRNA and target homolog pairs when searching all reference and representative genomes in RefSeq?
15	Number of sRNA:target homologs (ALL)	Y		Medium	Number of sRNA and target homolog pairs (max 100) when searching all reference and representative genomes in RefSeq

16	Number of sRNA homologs (16S relatives)	Y		Fast	Number of sRNA homologs (max 100) when searching 250 closest relatives as determined from 16S sequences
17	Number of target homologs (16S relatives)	Y		Fast	Number of target homologs (max 10) when searching 250 closest relatives as determined from 16S sequences
18	Are there sRNA:target homologs (16S relatives)?	Y		Fast	Are there any sRNA and target homolog pairs when searching 250 closest relatives as determined from 16S sequences?
19	Number of sRNA:target homologs (16S relatives)	Y		Fast	Number of sRNA and target homolog pairs (max 10) when searching 250 closest relatives as determined from 16S sequences
20	Seed of length 7 bps	Y		Fast	Is there a seed of length 7 nucleotides, i.e., 7 consecutive basepairs between the sRNA and target sequences
21	Seed of length 8 bps	Y		Fast	Is there a seed of length 8 nucleotides, i.e., 8 consecutive basepairs between the sRNA and target sequences
22	Seed of length 9 bps	Y		Fast	Is there a seed of length 9 nucleotides, i.e., 9 consecutive basepairs between the sRNA and target sequences
23	Seed of length 10 bps	Y		Fast	Is there a seed of length 10 nucleotides, i.e., 10 consecutive basepairs between the sRNA and target sequences
24	IntaRNA: is interaction found?	Y	IntaRNA	Medium	Did IntaRNA report an interaction?
25	IntaRNA: energy	Y	IntaRNA	Medium	Energy associated with IntaRNA interaction
26	IntaRNA: length of sRNA interacting region	Y	IntaRNA	Medium	Length of sRNA interacting region as reported by IntaRNA
27	IntaRNA: length of target interacting region	Y	IntaRNA	Medium	Length of target interacting region as reported by IntaRNA
28	IntaRNA: is interaction upstream of start codon?	Y	IntaRNA	Medium	Is interaction reported by IntaRNA upstream of the target start codon?
29	IntaRNA: distance of interaction from start codon	Y	IntaRNA	Medium	Distance in nucleotides from the start codon of the interaction reported by IntaRNA
30	IntaRNA: number of AU bps	Y	IntaRNA	Medium	Number of A:U basepairs in interaction reported by IntaRNA
31	IntaRNA: number of GC bps	Y	IntaRNA	Medium	Number of G:C basepairs in interaction reported by IntaRNA
32	IntaRNA: number of GU basepairs	Y	IntaRNA	Medium	Number of G:U basepairs in interaction reported by IntaRNA
33	IntaRNA: total number of bps	Y	IntaRNA	Medium	Number of all basepairs in interaction reported by IntaRNA
34	IntaRNA: number of stacking regions	Y	IntaRNA	Medium	Number of stacking regions in interaction reported by IntaRNA
35	IntaRNA: length of largest stacking region	Y	IntaRNA	Medium	Number of nucleotides in largest stacking region in interaction reported by IntaRNA
36	IntaRNA: length of largest canonical stacking region	Y	IntaRNA	Medium	Number of nucleotides in largest canonical stacking region in interaction reported by IntaRNA
37	IntaRNA: total length of interior loops	Y	IntaRNA	Medium	Number of nucleotides in all interior loops in interaction reported by IntaRNA
38	IntaRNA: number of interior loops	Y	IntaRNA	Medium	Number of interior loops in interaction reported by IntaRNA
39	IntaRNA: length of largest interior loop	Y	IntaRNA	Medium	Number of nucleotides in largest interior loop in interaction reported by IntaRNA
40	IntaRNA: total length of bulge loops	Y	IntaRNA	Medium	Number of nucleotides in all bulge loops in interaction reported by IntaRNA
41	IntaRNA: number of bulge loops	Y	IntaRNA	Medium	Number of bulge loops in interaction reported by IntaRNA
42	IntaRNA: length of largest bulge loop	Y	IntaRNA	Medium	Number of nucleotides in largest bulge loop in interaction reported by IntaRNA
43	CopraRNA: p-value	Y	CopraRNA	Slow	P-value of interaction reported by CopraRNA
44	CopraRNA: false-discovery-rate	Y	CopraRNA	Slow	False-discovery-rate of interaction reported by CopraRNA

45	Minimum free energy of interaction	Y		Medium	Minimum free energy of hybridization of sRNA and target
46	RNAup: sRNA accessibility energy	Y	RNAup	Slow	Accessibility energy of sRNA as reported by RNAup
47	RNAup: target accessibility energy	Y	RNAup	Slow	Accessibility energy of target as reported by RNAup
48	RNAup: interaction energy	Y	RNAup	Slow	Hybridization energy of sRNA and target as reported by RNAup
49	RNAup: total binding energy	Y	RNAup	Slow	Total binding energy of sRNA and target as reported by RNAup
50	RNAplex: energy not considering accessibility	Y	RNAplex	Fast	Hybridization energy of sRNA and target as reported by RNAplex
51	RNAplex: energy considering accessibility	Y	RNAplex	Fast	Total binding energy of sRNA and target as reported by RNAplex
52	sRNARFTarget: probability	Y	sRNARFTarget	Fast	Probability of sRNA and target interaction as reported by sRNARFTarget
53	AAA	Y	sRNARFTarget	Fast	Difference in frequency of trinucleotide between the target sequence and the sRNA sequence
54	AAC	Y	sRNARFTarget	Fast	Difference in frequency of trinucleotide between the target sequence and the sRNA sequence
55	AAG	Y	sRNARFTarget	Fast	Difference in frequency of trinucleotide between the target sequence and the sRNA sequence
56	AAT	Y	sRNARFTarget	Fast	Difference in frequency of trinucleotide between the target sequence and the sRNA sequence
57	ACA	Y	sRNARFTarget	Fast	Difference in frequency of trinucleotide between the target sequence and the sRNA sequence
58	ACC	Y	sRNARFTarget	Fast	Difference in frequency of trinucleotide between the target sequence and the sRNA sequence
59	ACG	Y	sRNARFTarget	Fast	Difference in frequency of trinucleotide between the target sequence and the sRNA sequence
60	ACT	Y	sRNARFTarget	Fast	Difference in frequency of trinucleotide between the target sequence and the sRNA sequence
61	AGA	Y	sRNARFTarget	Fast	Difference in frequency of trinucleotide between the target sequence and the sRNA sequence
62	AGC	Y	sRNARFTarget	Fast	Difference in frequency of trinucleotide between the target sequence and the sRNA sequence
63	AGG	Y	sRNARFTarget	Fast	Difference in frequency of trinucleotide between the target sequence and the sRNA sequence
64	AGT	Y	sRNARFTarget	Fast	Difference in frequency of trinucleotide between the target sequence and the sRNA sequence
65	ATA	Y	sRNARFTarget	Fast	Difference in frequency of trinucleotide between the target sequence and the sRNA sequence
66	ATC	Y	sRNARFTarget	Fast	Difference in frequency of trinucleotide between the target sequence and the sRNA sequence
67	ATG	Y	sRNARFTarget	Fast	Difference in frequency of trinucleotide between the target sequence and the sRNA sequence
68	ATT	Y	sRNARFTarget	Fast	Difference in frequency of trinucleotide between the target sequence and the sRNA sequence
69	CAA	Y	sRNARFTarget	Fast	Difference in frequency of trinucleotide between the target sequence and the sRNA sequence
70	CAC	Y	sRNARFTarget	Fast	Difference in frequency of trinucleotide between the target sequence and the sRNA sequence
71	CAG	Y	sRNARFTarget	Fast	Difference in frequency of trinucleotide between the target sequence and the sRNA sequence
72	CAT	Y	sRNARFTarget	Fast	Difference in frequency of trinucleotide between the target sequence and the sRNA sequence
73	CCA	Y	sRNARFTarget	Fast	Difference in frequency of trinucleotide between the target sequence and the sRNA sequence
74	CCC	Y	sRNARFTarget	Fast	Difference in frequency of trinucleotide between the target sequence and the sRNA sequence
75	CCG	Y	sRNARFTarget	Fast	Difference in frequency of trinucleotide between the target sequence and the sRNA sequence
76	CCT	Y	sRNARFTarget	Fast	Difference in frequency of trinucleotide between the target sequence and the sRNA sequence
77	CGA	Y	sRNARFTarget	Fast	Difference in frequency of trinucleotide between the target sequence and the sRNA sequence
78	CGC	Y	sRNARFTarget	Fast	Difference in frequency of trinucleotide between the target sequence and the sRNA sequence
79	CGG	Y	sRNARFTarget	Fast	Difference in frequency of trinucleotide between the target sequence and the sRNA sequence
80	CGT	Y	sRNARFTarget	Fast	Difference in frequency of trinucleotide between the target sequence and the sRNA sequence
81	CTA	Y	sRNARFTarget	Fast	Difference in frequency of trinucleotide between the target sequence and the sRNA sequence
82	CTC	Y	sRNARFTarget	Fast	Difference in frequency of trinucleotide between the target sequence and the sRNA sequence
83	CTG	Y	sRNARFTarget	Fast	Difference in frequency of trinucleotide between the target sequence and the sRNA sequence
84	CTT	Y	sRNARFTarget	Fast	Difference in frequency of trinucleotide between the target sequence and the sRNA sequence
85	GAA	Y	sRNARFTarget	Fast	Difference in frequency of trinucleotide between the target sequence and the sRNA sequence
86	GAC	Y	sRNARFTarget	Fast	Difference in frequency of trinucleotide between the target sequence and the sRNA sequence
87	GAG	Y	sRNARFTarget	Fast	Difference in frequency of trinucleotide between the target sequence and the sRNA sequence
88	GAT	Y	sRNARFTarget	Fast	Difference in frequency of trinucleotide between the target sequence and the sRNA sequence
89	GCA	Y	sRNARFTarget	Fast	Difference in frequency of trinucleotide between the target sequence and the sRNA sequence

90	GCC	Y	sRNARFTarget	Fast	Difference in frequency of trinucleotide between the target sequence and the sRNA sequence
91	GCG	Y	sRNARFTarget	Fast	Difference in frequency of trinucleotide between the target sequence and the sRNA sequence
92	GCT	Y	sRNARFTarget	Fast	Difference in frequency of trinucleotide between the target sequence and the sRNA sequence
93	GGA	Y	sRNARFTarget	Fast	Difference in frequency of trinucleotide between the target sequence and the sRNA sequence
94	GGC	Y	sRNARFTarget	Fast	Difference in frequency of trinucleotide between the target sequence and the sRNA sequence
95	GGG	Y	sRNARFTarget	Fast	Difference in frequency of trinucleotide between the target sequence and the sRNA sequence
96	GGT	Y	sRNARFTarget	Fast	Difference in frequency of trinucleotide between the target sequence and the sRNA sequence
97	GTA	Y	sRNARFTarget	Fast	Difference in frequency of trinucleotide between the target sequence and the sRNA sequence
98	GTC	Y	sRNARFTarget	Fast	Difference in frequency of trinucleotide between the target sequence and the sRNA sequence
99	GTG	Y	sRNARFTarget	Fast	Difference in frequency of trinucleotide between the target sequence and the sRNA sequence
100	GTT	Y	sRNARFTarget	Fast	Difference in frequency of trinucleotide between the target sequence and the sRNA sequence
101	TAA	Y	sRNARFTarget	Fast	Difference in frequency of trinucleotide between the target sequence and the sRNA sequence
102	TAC	Y	sRNARFTarget	Fast	Difference in frequency of trinucleotide between the target sequence and the sRNA sequence
103	TAG	Y	sRNARFTarget	Fast	Difference in frequency of trinucleotide between the target sequence and the sRNA sequence
104	TAT	Y	sRNARFTarget	Fast	Difference in frequency of trinucleotide between the target sequence and the sRNA sequence
105	TCA	Y	sRNARFTarget	Fast	Difference in frequency of trinucleotide between the target sequence and the sRNA sequence
106	TCC	Y	sRNARFTarget	Fast	Difference in frequency of trinucleotide between the target sequence and the sRNA sequence
107	TCG	Y	sRNARFTarget	Fast	Difference in frequency of trinucleotide between the target sequence and the sRNA sequence
108	TCT	Y	sRNARFTarget	Fast	Difference in frequency of trinucleotide between the target sequence and the sRNA sequence
109	TGA	Y	sRNARFTarget	Fast	Difference in frequency of trinucleotide between the target sequence and the sRNA sequence
110	TGC	Y	sRNARFTarget	Fast	Difference in frequency of trinucleotide between the target sequence and the sRNA sequence
111	TGG	Y	sRNARFTarget	Fast	Difference in frequency of trinucleotide between the target sequence and the sRNA sequence
112	TGT	Y	sRNARFTarget	Fast	Difference in frequency of trinucleotide between the target sequence and the sRNA sequence
113	TTA	Y	sRNARFTarget	Fast	Difference in frequency of trinucleotide between the target sequence and the sRNA sequence
114	TTC	Y	sRNARFTarget	Fast	Difference in frequency of trinucleotide between the target sequence and the sRNA sequence
115	TTG	Y	sRNARFTarget	Fast	Difference in frequency of trinucleotide between the target sequence and the sRNA sequence
116	TTT	Y	sRNARFTarget	Fast	Difference in frequency of trinucleotide between the target sequence and the sRNA sequence
117	TargetRNA3: probability		TargetRNA3	Fast	Probability of sRNA and target interaction as reported by TargetRNA3
118	Evinced Interaction				Is there experimental evidence for an interaction between the sRNA and target?

Table S4.

The table indicates the performance of 8 different machine learning algorithms using different measures: area under the ROC curve (AUC), F1 score (F1), Matthews correlation coefficient (MCC), sensitivity at a false positive rate of 5%, and the time in seconds required for training the machine learning model.

Algorithm	AUC	F1	MCC	Sensitivity at 5% False Positive Rate	Training Time (in seconds)
Gradient Boosting	0.75	0.35	0.28	0.22	1
Random Forest	0.74	0.32	0.25	0.21	1
Neural Network	0.72	0.21	0.18	0.16	6
Quadratic Discriminant Analysis	0.68	0.39	0.21	0.11	0
Gaussian Naive Bayes	0.67	0.39	0.22	0.11	0
k Nearest Neighbors	0.66	0.36	0.20	0.13	0
Logistic Regression	0.65	0.16	0.15	0.13	0
Support Vector Machine	0.65	0.18	0.23	0.15	48

Table S5.

The table indicates the performance of 6 different tools for predicting targets of sRNA regulation.

Tool	AUC	Sensitivity at 5% False Positive Rate	Average Runtime per sRNA (minutes)
TargetRNA3	0.68	0.18	< 1
CopraRNA	0.64	0.16	189
RNAup	0.59	0.11	47
IntaRNA	0.57	0.08	3.5
sRNARFTarget	0.56	0.06	< 1
RNAplex	0.55	0.06	< 1
