# Supplementary material for: TargetRNA3: predicting prokaryotic RNA regulatory targets with machine learning
Source: Genome Biol. 2023 Dec 1;24:276. doi: 10.1186/s13059-023-03117-2 (PMC10691042; doi:10.1186/s13059-023-03117-2)
Supplement: Supplementary file 2 — Additional file 2. Peer review history. [file 13059_2023_3117_MOESM2_ESM.docx]

**Review history**

**First round of review**

**Reviewer 1**

-Is the Software likely to be of broad utility? Is it easy to install and use?

Yes. The software is addressing a challenging area in microbiology and could be of utility. I didn't install the software from source code but I did try the web version. There are two main issues with the web version that would make the software less useful: 1) If the sRNA sequence is given in FASTA format with a NCBI header then it returns a not-very-friendly error (Internal Server Error: Missing headers or other script malfunction. Check the logs or contact cs-sysadmin@wellesley.edu) - also the web site doesn't say what characters are allowed in the header; 2) If the software doesn't find a significant target with the default sensitivity it is not possible to see the results for all the mRNAs on that genome and the user needs to go back and forth until a sensitivity threshold that returns some results is found.


-Does the software represent a significant advance over previously published tools (as demonstrated by direct comparison with available related software)?

The software does report a slightly higher AUROC than other related software, and it is faster than the best related software.


-Please indicate briefly the novel features and/or advantages of the software and/or please reference the relevant publications and which alternatives, if any, it should be compared with.

It uses a larger data set for training/testing, achieves higher AUROC than related software on a partition of this data set, and is fast.


-Are the methods appropriate to the aims of the study, are they well described, and are necessary controls included?
-Are the conclusions adequately supported by the data shown?
-Are sufficient details provided to allow replication and comparison with related analyses that may have been performed?

The best practice in machine leaning is to use cross-validation to evaluate the performance of a classifier instead of using a single training/testing partition. Furthermore, it is better to leave completely independent data for further assessing of the model. By randomly splitting the data, it is likely that a sRNA might be in both the training and testing partition of the data just with a different target. Furthermore to show that the model is able to generalize to unseen data it would be recommendable to leave out an organism from the training process; otherwise, it is possible that data leaking is happening and the reported performance is over-optimistic.

The following is unclear or missing:
1) how the p-value associated with a sRNA:target interaction is calculated.
2) what sequences are considered seeds and how they are found
3) a summary table of the data listing the 13 bacterial genomes used and the number of sRNAs and targets per genome.
4) a table with the actual times shown in Figure 5(C).
5) what correlation is used for Figure 2(B)? Pearson? Spearman?

There is also a contradiction in the manuscript: In Figure 2 the "Distance to upstream gene from target start codon" is listed which indicates this is an useful feature; however, in lines 114 and 194 is said that "the distance between a possible target and its upstream gene does not contain substantial predictive power" so why was this feature included in TargetRNA3?

It makes sense to run the related software with default parameter settings; however, related software should be run as it was meant to be run. In line 290 says that the target interaction was searched from "200 nucleotides upstream to 100 nucleotides downstream of the start of the target"; however, for example, sRNARFTarget expects as input the complete target (mRNA) sequence so providing to this software a different type of sequence is incorrect and likely affects its performance. So, the author should verify that each of the related software included in the comparison is run with the input that software expects.

Finally, the manuscript could be greatly improved by doing a feature importance analysis. What features are the most relevant for Gradient boosting? How each feature contributes to make a prediction? Does the probability of being a target increases or decreases with the distance? Does the probability of being a target increases or decreases with the number of homologs? Using SHAP could provide some insights on what type of targets are being predicted.


-Is the paper of broad interest to others in the field, or of outstanding interest to a broad audience of biologists?

The paper would be of interest to microbiologists.

**Reviewer 2**

Tjaden presents an updated method for predicting bacterial sRNA:mRNA, TargetRNA3.

Overall the manuscript is well written, and the methods appear to be
thorough. Although I have some suggestions below. I also ran several
predictions for a negative and positive set of ncRNAs for E. coli
K-12.

I ran a simple test of TargetRNA3 and found the webserver to be
intuitive and handy to use. However, I also noted a number of
issues. On my "negative" set of sRNAs that are unlikely to form
functional interactions with mRNAs which include csrB, 6S, and
tRNA-Ser I found a number of surprising interactions in the
predictions, all of which are unlikely. The P-values for these are far
too low in my opinion, it seems likely that these have not been
corrected for multiple testing, if this were to be done, then I
suspect the results would be more reasonable (sequences & top 5
predictions are provided below).

For the positive set of sRNAs there were some additional issues. Many
likely not of the author's making. I selected sok, DsrA, sroB and
OxyS. Based on Rfam descriptions these should each interact with
mRNAs. For 'sok' the expected interaction with 'hok' was not predicted
-- many short toxins are missed from genome annotations. For the other
3 sRNAs I did not see any of the expected mRNAs in the results lists
either. This could be due to aliases/synonyms being used, perhaps
these could be listed in the table too, when known?

The OxyS:OxyR prediction is interesting. I did a very quick literature
search and saw several references to the oxyR transcriptional
regulator activating OxyS transcription, but no obvious mention of OxyS
regulating OxyR translation.

1. The term 'evinced' seems unnecessary and is over-used in the manuscript IMHO.

2. IntaRNA is mentioned in the introduction, but not RNAup. My
understanding is the IntaRNA is a clone of RNAup with some minor speed
improvements.

3. A "large set of experimentally evinced sRNA:target interactions" is
used for training and testing. It is worth acknowledging that
experimentally identified interactions are likely to include a number
of interactions that have little impact on fitness, i.e. are
stochastic and non-functional. How many of the 4,386 interactions have
evidence that they are maintained by selection/are conserved?

4. Of the 13 genomes, how many of the major bacterial phyla/orders are represented?

5. The sentence "Altogether then, we compiled data on 329,548 pairs of
sRNAs and possible targets, 4,386 of which are evinced interactions
and 325,162 of which are considered non-interactions." is redundant.

6. What does "highly significant" mean?

7. The comparison of ML techniques is interesting. Difficult to judge
from the figure which method "wins". Could summary statistics lie
AUROC, MCC, F1, ... and walltime be given to justify the selection of
gradient boosting?

8. It may be worth mentioning benchmarks in addition to Pain (2015),
e.g. Lai & Meyer (2015) and Umu & Gardner (2017). Although I
acknowledge these are more general than the bacterial sRNA:mRNA
problem.

9. It is unclear why CsrA is mentioned on pg 13. While it does bind
ncRNA & mRNAs, generally sRNA:mRNA interacitons are not involved.

10. For biological sequences random splits into training and test data
is generally a very bad idea. Homology between sequences can inflate
the assements of accuracy. The author should at a minimum check
whether this is an issue in this case, and ideally correct the
training if so.

11. Figures 1 & 2 are unreadable, on both the printout and
on-screen. Perhaps these should be tables instead? Sorting Figure 1 on
F-statistic also seems like it would help the reader. Figure 5 is
pixellated too.

12. Could AUROC be added to the legend for Figures 3 & 4?

######################################################################
#Negative set:
CsrB
>U00096.3:c2924515-2924156 Escherichia coli str. K-12 substr. MG1655, complete genome
GAGTCAGACAACGAAGTGAACATCAGGATGATGACACTTCTGCAGGACACACCAGGATGGTGTTTCAGGG
AAAGGCTTCTGGATGAAGCGAAGAGGATGACGCAGGACGCGTTAAAGGACACCTCCAGGATGGAGAATGA
GAACCGGTCAGGATGATTCGGTGGGTCAGGAAGGCCAGGGACACTTCAGGATGAAGTATCACATCGGGGT
GGTGTGAGCAGGAAGCAATAGTTCAGGATGAACGATTGGCCGCAAGGCCAGAGGAAAAGTTGTCAAGGAT
GAGCAGGGAGCAACAAAAGTAGCTGGAATGCTGCGAAACGAACCGGGAGCGCTGTGAATACAGTGCTCCC
TTTTTTTATT

Number of predicted targets: 22

Rank Target Energy p-value Probability
1 mraZ (b0081) -23.67 8.1e-05 0.61
2 ypjA (b2647) -17.06 2.7e-04 0.58
3 alaA (b2290) -23.42 6.0e-04 0.56
4 tnaC (b3707) -19.59 6.4e-04 0.56
5 dnaA (b3702) -24.58 6.7e-04 0.56

U00096 CMSEARCH misc_RNA 3055983 3056166 129.5 + . UID=misc_RNA.10096;E-value=1.5e-28;Name=6S;
>U00096; Escherichia coli str. K-12 substr. MG1655, complete genome.
atttctctgagatgttcgcaagcgggccagtcccctgagccgatatttca
taccacaagaatgtggcgctccgcggttggtgagcatgctcggtccgtcc
gagaagccttaaaactgcgacgacacattcaccttgaaccaagggttcaa
gggttacagcctgcggcggcatctcggagattcc

Number of predicted targets: 9

Rank Target Energy p-value Probability
1 yghJ (b4466) -9.75 4.2e-06 0.64
2 ypjA (b2647) -10.31 1.0e-05 0.62
3 mraZ (b0081) -20.55 9.8e-05 0.57
4 shoB (b4687) -17.67 2.1e-04 0.55
5 oppA (b1243) -12.63 2.6e-04 0.55

U00096 tRNAscan131 tRNA 925884 925971 76.75 - . number=81;type=Ser;anticodon=GGA;
>U00096; Escherichia coli str. K-12 substr. MG1655, complete genome.
ggtgaggtgtccgagtggctgaaggagcacgcctggaaagtgtgtatacg
gcaacgtatcgggggttcgaatccccccctcaccgcca

Number of predicted targets: 7

Rank Target Energy p-value Probability
1 mtfA (b1976) -41.43 6.1e-08 0.71
2 ypjA (b2647) -13.08 1.7e-05 0.61
3 ompF (b0929) -13.59 1.5e-04 0.56
4 mraZ (b0081) -13.43 3.4e-04 0.54
5 cadC (b4133) -12.91 4.9e-04 0.53

######################################################################
#Positive set:
U00096 CMSEARCH misc_RNA 16901 17053 123.5 - . UID=misc_RNA.10211;E-value=4.9e-28;Name=sok;
Expected interactions with mRNA for the hok toxin

>U00096; Escherichia coli str. K-12 substr. MG1655, complete genome.
gggtgcttgaggctgtctgtctcaggcattagctgaacggcagatagaga
aaagccccgagtgatattttaccatcaacccgaggcctcctatatgctga
acacatgtagagtgcctcttactgaccgtaaggtcaaggagaagagagca
atg

Number of predicted targets: 10
Rank Target Energy p-value Probability
1 yghJ (b4466) -11.49 1.1e-06 0.66
2 ypjA (b2647) -12.34 2.6e-06 0.64
3 ivbL (b3672) -23.72 1.0e-04 0.56
4 cadC (b4133) -18.44 2.5e-04 0.54
5 ompF (b0929) -11.26 3.3e-04 0.54

U00096 CMSEARCH misc_RNA 2025227 2025313 102.8 - . UID=misc_RNA.10097;E-value=3.4e-20;Name=DsrA;
Expected interactions with RpoS, possibly hns, rbsD, argR, ilvI

>U00096; Escherichia coli str. K-12 substr. MG1655, complete genome.
aacacatcagatttcctggtgtaacgaattttttaagtgcttcttgctta
agcaagtttcatcccgaccccctcagggtcgggattt

Number of predicted targets: 8
Rank Target Energy p-value Probability
1 yghJ (b4466) -10.49 1.9e-07 0.64
2 mraZ (b0081) -17.75 1.5e-05 0.57
3 ompF (b0929) -12.21 5.0e-05 0.55
4 ypjA (b2647) -7.82 1.1e-04 0.53
5 oppA (b1243) -11.54 1.4e-04 0.52

U00096 CMSEARCH misc_RNA 507204 507287 83.0 + . UID=misc_RNA.10155;E-value=3.1e-12;Name=sroB;
Expected interactions with YbfM, DpiA/DpiB, chbBC intergenic region.
>U00096; Escherichia coli str. K-12 substr. MG1655, complete genome.
acaccgtcgcttaaagtgacggcataataataaaaaaatgaaattcctct
ttgacgggccaatagcgatattggccattttttt

Number of predicted targets: 6
Rank Target Energy p-value Probability
1 ybaK (b0481) -99.5 3.8e-09 0.72
2 yghJ (b4466) -11.05 1.6e-07 0.66
3 ypjA (b2647) -11.82 4.4e-07 0.64
4 ykiD (b4732) -17.31 6.8e-05 0.55
5 mraZ (b0081) -11.74 1.7e-04 0.53

OxyS RNA
Expected interactions with oxyR, yobF, ybaY

>U00096.3:c4158394-4158285 Escherichia coli str. K-12 substr. MG1655, complete genome
GAAACGGAGCGGCACCTCTTTTAACCCTTGAAGTCACTGCCCGTTTCGAGAGTTTCTCAACTCGAATAAC
TAAAGCCAACGTGAACTTTTGCGGATCTCCAGGATCCGCT

Number of predicted targets: 10

Rank Target Energy p-value Probability
1 oxyR (b3961) -151.82 5.9e-09 0.72
2 yghJ (b4466) -8.64 1.0e-06 0.63
3 mreB (b3251) -10.42 1.6e-06 0.63
4 ypjA (b2647) -8.73 4.6e-06 0.61
5 eutJ (b2454) -8.11 1.3e-05 0.59

**Authors’ response to reviewers**

We sincerely thank the reviewers for their rigorous evaluation, for noting our contributions, and for their constructive feedback. They raised excellent points. We believe our revisions in response to the reviewers' careful feedback have strengthened the manuscript significantly. As requested, we have provided a detailed response to each reviewer comment below and revisions in the updated manuscript have been marked (in red). As examples, major revisions include the following:

• Reviewers astutely observed that the "testing" data we used to evaluate performance of TargetRNA3 may not be independent from the "training" data we used to build the model, thereby biasing results. As the reviewers recommended, we have addressed this by using "testing" data to evaluate performance that comes strictly from different genomes than those used in our "training" data.

• We performed a new analysis, as suggested by reviewers, to better elucidate the relative contribution of different features toward distinguishing targets from non-targets. We implemented a SHAP analysis, as advised, which enables global measures of feature impact on a machine learning model.


*****************************************************************************
Reviewer #1:

-Is the Software likely to be of broad utility? Is it easy to install and use?

Yes. The software is addressing a challenging area in microbiology and could be of utility. I didn't install the software from source code but I did try the web version. There are two main issues with the web version that would make the software less useful: 1) If the sRNA sequence is given in FASTA format with a NCBI header then it returns a not-very-friendly error (Internal Server Error: Missing headers or other script malfunction. Check the logs or contact cs-sysadmin@wellesley.edu) - also the web site doesn't say what characters are allowed in the header; 2) If the software doesn't find a significant target with the default sensitivity it is not possible to see the results for all the mRNAs on that genome and the user needs to go back and forth until a sensitivity threshold that returns some results is found.

Thank you for bringing these issues to our attention.
1) The input process has been fixed so that FASTA formatted input with any headers, including NCBI headers, are accepted and there are no restrictions on what characters are allowed in the header.
2) When the software finds no statistically significant targets, we now provide a clearer message indicating this as well as instructions for seeing the results of all predictions (significant or not), obviating the need for the user to go back and forth running the software. We experimented with showing all predictions (by default) in the case when the software finds no significant targets, but had concerns that some less experienced users might have confusion about whether the results returned by the software were significant or not. We believe our revised output is a judicious compromise.


*****************************************************************************
-Does the software represent a significant advance over previously published tools (as demonstrated by direct comparison with available related software)?

The software does report a slightly higher AUROC than other related software, and it is faster than the best related software.

Thank you.


*****************************************************************************
-Please indicate briefly the novel features and/or advantages of the software and/or please reference the relevant publications and which alternatives, if any, it should be compared with.

It uses a larger data set for training/testing, achieves higher AUROC than related software on a partition of this data set, and is fast.

Thank you.


*****************************************************************************
-Are the methods appropriate to the aims of the study, are they well described, and are necessary controls included?

-Are the conclusions adequately supported by the data shown?

-Are sufficient details provided to allow replication and comparison with related analyses that may have been performed?

The best practice in machine leaning is to use cross-validation to evaluate the performance of a classifier instead of using a single training/testing partition. Furthermore, it is better to leave completely independent data for further assessing of the model. By randomly splitting the data, it is likely that a sRNA might be in both the training and testing partition of the data just with a different target. Furthermore to show that the model is able to generalize to unseen data it would be recommendable to leave out an organism from the training process; otherwise, it is possible that data leaking is happening and the reported performance is over-optimistic.

An excellent point! We completely agree. And we apologize that our initial manuscript submission was not clear about how we partitioned the data. Previously, we partitioned training/testing data by sRNAs so there was no overlap between sRNAs in testing data and sRNAs in training data, but we did not take organisms into account. In response to the reviewers' prudent feedback, we now consider organisms when partitioning, so that there is no overlap between organisms in testing data and organisms in training data. Based on the training data, 5-fold cross validation is used to evaluate different machine learning algorithms and tune hyperparameters. Finally, testing data is used to evaluate TargetRNA3's performance in comparison to existing tools for predicting sRNA regulatory targets. Details are provided in the Methods section.


*****************************************************************************
The following is unclear or missing:

1) how the p-value associated with a sRNA:target interaction is calculated.

We have revised the Methods section to better describe the p-value calculation. In short, a log-normal distribution is fit to the interaction probabilities for a given sRNA, and p¬-values are computed from the cumulative distribution function (CDF) of the fitted log-normal distribution.


*****************************************************************************
2) what sequences are considered seeds and how they are found

Seeds are regions of consecutive basepairs (often 7-10 bps in length) in the sRNA and mRNA that are perfectly complementary. This has been more carefully described in the Results section and in the Supplementary Material.


*****************************************************************************
3) a summary table of the data listing the 13 bacterial genomes used and the number of sRNAs and targets per genome.

Good point. We have now included such a table (Supplementary Table 4).


*****************************************************************************
4) a table with the actual times shown in Figure 5(C).

A table with these data has been added (Supplementary Table 5).


*****************************************************************************
5) what correlation is used for Figure 2(B)? Pearson? Spearman?

We apologize for the omission. This is Pearson correlation. We have updated the Figure legend to specify this (and note that the former Figure 2 is now named Supplementary Figure 3 because we have newly incorporated a SHAP analysis, as helpfully advised by this reviewer, which better illuminates feature importance as Figure 2).


*****************************************************************************
There is also a contradiction in the manuscript: In Figure 2 the "Distance to upstream gene from target start codon" is listed which indicates this is an useful feature; however, in lines 114 and 194 is said that "the distance between a possible target and its upstream gene does not contain substantial predictive power" so why was this feature included in TargetRNA3?

Thank you for noting this. We agree that it is confusing language. We have removed the seemingly contradictory sentences, "the distance between a possible target and its upstream gene does not contain substantial predictive power." Our intent was to characterize the feature as having "weaker" rather than "stronger" predictive power, but we were careless in not defining descriptive terms such as "weaker" or "stronger" or "substantial." As mentioned, for these two particular sentences, we removed them. Elsewhere in the manuscript, we revised it to be more precise, referencing p-values and defining terms.


*****************************************************************************
It makes sense to run the related software with default parameter settings; however, related software should be run as it was meant to be run. In line 290 says that the target interaction was searched from "200 nucleotides upstream to 100 nucleotides downstream of the start of the target"; however, for example, sRNARFTarget expects as input the complete target (mRNA) sequence so providing to this software a different type of sequence is incorrect and likely affects its performance. So, the author should verify that each of the related software included in the comparison is run with the input that software expects.

We thank the reviewer for this great point. For consistency in comparison, we ran all tools with the same target input sequences, i.e., from 200 nucleotides upstream to 100 nucleotides downstream of the start of the target. These are the sequences used by the authors of CopraRNA and of IntaRNA (at least for the most recent version of IntaRNA, version 2 from 2017 that we used in this study, although the original version, version 1 from 2008, used 150 nucleotides upstream to 50 nucleotides downstream). These are also the sequences used in assessments by other groups (Pain et al. 2015, Uma & Gardner 2017). As RNAup and RNAplex weren't designed specifically for prokaryotic sRNAs, the authors do not provide guidance on input sequences in this context. However, for sRNARFTarget, the random forest model developed by the authors was trained on the complete target (mRNA) sequence, as the reviewer astutely notes. We agree that a fairer comparison would be to execute sRNARFTarget on similar sequences to which it was trained. We thank the reviewer for pointing this out. We have executed sRNARFTarget using complete target sequences, as advised. Doing so improved the performance of sRNARFTarget. We have updated the results accordingly and described these details in the Methods section.


*****************************************************************************
Finally, the manuscript could be greatly improved by doing a feature importance analysis. What features are the most relevant for Gradient boosting? How each feature contributes to make a prediction? Does the probability of being a target increases or decreases with the distance? Does the probability of being a target increases or decreases with the number of homologs? Using SHAP could provide some insights on what type of targets are being predicted.

We are sincerely grateful for this suggestion. As recommended, we performed a SHAP analysis to better understand the importance of different features employed by TargetRNA3 (see Figure 2 and Results section). The SHAP analysis illuminates each feature's impact on the model's output. Two of the most impactful features are the hybridization energy of the two RNAs (as determined by RNAplex) and the level of conservation (as reflected by the number of sRNA:target homologs). Larger negative hybridization energies and higher levels of conservation increase the probability of a target. Supplementary Figure 3 provides a supplemental perspective to the SHAP analysis on the relationship of interactions to the different features.


*****************************************************************************
-Is the paper of broad interest to others in the field, or of outstanding interest to a broad audience of biologists?

The paper would be of interest to microbiologists.

Thank you.


*****************************************************************************
Reviewer #2:

Tjaden presents an updated method for predicting bacterial sRNA:mRNA, TargetRNA3.

Overall the manuscript is well written, and the methods appear to be thorough. Although I have some suggestions below.

Thank you.


*****************************************************************************
I also ran several predictions for a negative and positive set of ncRNAs for E. coli K-12. I ran a simple test of TargetRNA3 and found the webserver to be intuitive and handy to use. However, I also noted a number of issues. On my "negative" set of sRNAs that are unlikely to form functional interactions with mRNAs which include csrB, 6S, and tRNA-Ser I found a number of surprising interactions in the predictions, all of which are unlikely. The P-values for these are far too low in my opinion, it seems likely that these have not been corrected for multiple testing, if this were to be done, then I suspect the results would be more reasonable (sequences & top 5 predictions are provided below).

For the positive set of sRNAs there were some additional issues. Many likely not of the author's making. I selected sok, DsrA, sroB and OxyS. Based on Rfam descriptions these should each interact with mRNAs. For 'sok' the expected interaction with 'hok' was not predicted -- many short toxins are missed from genome annotations. For the other 3 sRNAs I did not see any of the expected mRNAs in the results lists either. This could be due to aliases/synonyms being used, perhaps these could be listed in the table too, when known?

We appreciate the reviewer providing these results and insights - they raise many important questions! Perhaps chief among the questions, as we understand them, are (1) is TargetRNA3 predicting all interactions (including those without function/fitness implications) or is TargetRNA3 predicting only *regulatory* interactions? and (2) how accurate are TargetRNA3's predictions?

In regard to (1), TargetRNA3 is almost certainly predicting non-functional interactions as well as regulatory interactions. Most of the experimental approaches for global detection of sRNA targets, e.g., RIL-seq and CLASH, capture RNAs that a sRNA interacts with, but not necessarily that it functionally regulates. Many of the 4000+ interactions used to train TargetRNA3 stem from these global interaction methods. As an example provided by the reviewer, csrB is unlikely to form functional interactions with mRNAs, yet in a CLASH experiment (Iosub et al. 2020), csrB interacted with several mRNAs, including two of the mRNAs (dnaA and oppA) predicted by TargetRNA3. We consider these two (dnaA and oppA) as "true positive" predictions because there is experimental evidence of interaction, though there may not be regulatory effects. The distinction between interactions and regulatory effects is critical, and we appreciate that this should have be more clearly conveyed in the manuscript. We thank the reviewer for raising this issue. We have revised the manuscript (and especially the penultimate paragraph of the Discussion section) to emphasize these points.

In regard to (2) and the accuracy of TargetRNA3, the 7 example sRNAs provided by the reviewer (3 negative and 4 positive) offer an interesting perspective. On the one hand, TargetRNA3 predicts between 6 and 22 interactions (out of ~4300 possible targets in E. coli) per sRNA, corresponding to a false positive rate of under 1%, which we consider to be very good. On the other hand, its sensitivity is low in that it does not identify a number of well studied regulatory interactions. Of the 11 interactions mentioned by the reviewer for the 4 positive sRNAs, TargetRNA3 identifies 1 of them (dsrA:hns). If TargetRNA3's false positive rate is raised from less than 1% to 5% then TargetRNA3 identifies 5 of the 11 mentioned interactions (sroB:dpiB, sroB:chbB, dsrA:argR, dsrA:hns, oxyS:yobF), for a sensitivity of 45% on this example set. We believe these results are consistent with those we found using a larger data set to evaluate TargetRNA3 (Figure 5A). These examples that the reviewer savvily offers highlight the fact that TargetRNA3 sensitivity, while better than that of other tools, is still low, as it is far from capturing all interactions. We have updated the manuscript (particularly the Discussion section) to communicate this more explicitly.

The reviewer also mentions listing aliases/synonyms in the table, which we think is an excellent suggestion. Unfortunately, the genomes and their annotations that we use from RefSeq/GenBank do not include aliases, beyond what we already include in the table. We investigated incorporating aliases from select (well-studied) genomes, such as E. coli, but opted to restrict our datasources to those that scale for prokaryotic genomes broadly. As a compromise, for each gene predicted as a target by TargetRNA3, we have provided a hyperlink to NCBI's webpage for that gene so that the user can quickly discern more information about a target of interest.


*****************************************************************************
The OxyS:OxyR prediction is interesting. I did a very quick literature search and saw several references to the oxyR transcriptional regulator activating OxyS transcription, but no obvious mention of OxyS regulating OxyR translation.

Interesting, indeed! We are not aware of studies showing OxyS regulating OxyR, so we consider it a false positive prediction, but it seems worthy of further investigation.


*****************************************************************************
1. The term 'evinced' seems unnecessary and is over-used in the manuscript IMHO.

We agree. The use of the term "evinced" has been scaled back substantially. Thanks!


*****************************************************************************
2. IntaRNA is mentioned in the introduction, but not RNAup. My understanding is the IntaRNA is a clone of RNAup with some minor speed improvements.

Good point. We have included a description of RNAup in the introduction where we discuss related work.


*****************************************************************************
3. A "large set of experimentally evinced sRNA:target interactions" is used for training and testing. It is worth acknowledging that experimentally identified interactions are likely to include a number of interactions that have little impact on fitness, i.e. are stochastic and non-functional. How many of the 4,386 interactions have evidence that they are maintained by selection/are conserved?

An insightful observation! A number of the interactions likely do not correspond to "regulatory targets" but other types of interactions, such as sponges, or may have little impact on fitness and be non-functional, as the reviewer notes. This is an important point and we have emphasized this in the revised manuscript.
In regard to how many of the 4,386 interactions are conserved, this is a fantastic yet difficult question. For the 4,386 interactions, we find that both the sRNA and target genes are together conserved in related prokaryotes 95% of the time, but is the interaction conserved? To our knowledge, few conserved interactions have been verified, and it is not clear that the same regions of the sRNA and target are necessarily interacting, so searching for compensatory mutations in the interactions may not be effective. We believe the conservation of interactions remains a major open question.


*****************************************************************************
4. Of the 13 genomes, how many of the major bacterial phyla/orders are represented?

We thank the reviewer for raising this question. We have included a new table (Supplementary Table 1) with this information.


*****************************************************************************
5. The sentence "Altogether then, we compiled data on 329,548 pairs of sRNAs and possible targets, 4,386 of which are evinced interactions and 325,162 of which are considered non-interactions." is redundant.

This redundant sentence has been removed.


*****************************************************************************
6. What does "highly significant" mean?

We agree that this term is not well defined. We have removed this description of the p-values. Also, at this reviewer's suggestion, we sorted the values in Figure 1 so it is more obvious visually which features have smaller or larger p-values.


*****************************************************************************
7. The comparison of ML techniques is interesting. Difficult to judge from the figure which method "wins". Could summary statistics lie AUROC, MCC, F1, ... and walltime be given to justify the selection of gradient boosting?

Thanks! We have included these statistics in a new table (Supplementary Table 4).


*****************************************************************************
8. It may be worth mentioning benchmarks in addition to Pain (2015), e.g. Lai & Meyer (2015) and Umu & Gardner (2017). Although I acknowledge these are more general than the bacterial sRNA:mRNA problem.

Thank you. We have included both of these studies.


*****************************************************************************
9. It is unclear why CsrA is mentioned on pg 13. While it does bind ncRNA & mRNAs, generally sRNA:mRNA interacitons are not involved.

A fair point. We have removed mention of CsrA here.


*****************************************************************************
10. For biological sequences random splits into training and test data is generally a very bad idea. Homology between sequences can inflate the assements of accuracy. The author should at a minimum check whether this is an issue in this case, and ideally correct the training if so.

A terrific suggestion! This was raised by both reviewers and with good reason. As mentioned above, we apologize that our initial manuscript submission was not clear about how we partitioned the data. Previously, we partitioned training/testing data by sRNAs so there was no overlap between sRNAs in testing data and sRNAs in training data, but we did not take organisms into account. In response to the reviewers' prudent feedback, we now consider organisms when partitioning, so that there is no overlap between organisms in testing data and organisms in training data. Based on the training data, 5-fold cross validation is used to evaluate different machine learning algorithms and tune hyperparameters. Finally, testing data is used to evaluate TargetRNA3's performance in comparison to existing tools for predicting sRNA regulatory targets. Details are provided in the Methods section.


*****************************************************************************
11. Figures 1 & 2 are unreadable, on both the printout and on-screen. Perhaps these should be tables instead? Sorting Figure 1 on F-statistic also seems like it would help the reader. Figure 5 is pixellated too.

We have fixed the unreadability and the pixelation of the figures (we believe it was an artifact of submission upload, and we apologize). We sorted Figure 1 as suggested and agree that it enhances its interpretability.


*****************************************************************************
12. Could AUROC be added to the legend for Figures 3 & 4?

We added AUROC as suggested (now named Figures 2 & 3 rather than 3 & 4), thanks.


######################################################################
#Negative set:
CsrB
>U00096.3:c2924515-2924156 Escherichia coli str. K-12 substr. MG1655, complete genome
GAGTCAGACAACGAAGTGAACATCAGGATGATGACACTTCTGCAGGACACACCAGGATGGTGTTTCAGGG
AAAGGCTTCTGGATGAAGCGAAGAGGATGACGCAGGACGCGTTAAAGGACACCTCCAGGATGGAGAATGA
GAACCGGTCAGGATGATTCGGTGGGTCAGGAAGGCCAGGGACACTTCAGGATGAAGTATCACATCGGGGT
GGTGTGAGCAGGAAGCAATAGTTCAGGATGAACGATTGGCCGCAAGGCCAGAGGAAAAGTTGTCAAGGAT
GAGCAGGGAGCAACAAAAGTAGCTGGAATGCTGCGAAACGAACCGGGAGCGCTGTGAATACAGTGCTCCC
TTTTTTTATT

Number of predicted targets: 22

Rank Target Energy p-value Probability
1 mraZ (b0081) -23.67 8.1e-05 0.61
2 ypjA (b2647) -17.06 2.7e-04 0.58
3 alaA (b2290) -23.42 6.0e-04 0.56
4 tnaC (b3707) -19.59 6.4e-04 0.56
5 dnaA (b3702) -24.58 6.7e-04 0.56

U00096 CMSEARCH misc_RNA 3055983 3056166 129.5 + . UID=misc_RNA.10096;E-value=1.5e-28;Name=6S;
>U00096; Escherichia coli str. K-12 substr. MG1655, complete genome.
atttctctgagatgttcgcaagcgggccagtcccctgagccgatatttca
taccacaagaatgtggcgctccgcggttggtgagcatgctcggtccgtcc
gagaagccttaaaactgcgacgacacattcaccttgaaccaagggttcaa
gggttacagcctgcggcggcatctcggagattcc

Number of predicted targets: 9
Rank Target Energy p-value Probability
1 yghJ (b4466) -9.75 4.2e-06 0.64
2 ypjA (b2647) -10.31 1.0e-05 0.62
3 mraZ (b0081) -20.55 9.8e-05 0.57
4 shoB (b4687) -17.67 2.1e-04 0.55
5 oppA (b1243) -12.63 2.6e-04 0.55

U00096 tRNAscan131 tRNA 925884 925971 76.75 - . number=81;type=Ser;anticodon=GGA;
>U00096; Escherichia coli str. K-12 substr. MG1655, complete genome.
ggtgaggtgtccgagtggctgaaggagcacgcctggaaagtgtgtatacg
gcaacgtatcgggggttcgaatccccccctcaccgcca

Number of predicted targets: 7
Rank Target Energy p-value Probability
1 mtfA (b1976) -41.43 6.1e-08 0.71
2 ypjA (b2647) -13.08 1.7e-05 0.61
3 ompF (b0929) -13.59 1.5e-04 0.56
4 mraZ (b0081) -13.43 3.4e-04 0.54
5 cadC (b4133) -12.91 4.9e-04 0.53

######################################################################
#Positive set:
U00096 CMSEARCH misc_RNA 16901 17053 123.5 - . UID=misc_RNA.10211;E-value=4.9e-28;Name=sok;
Expected interactions with mRNA for the hok toxin

>U00096; Escherichia coli str. K-12 substr. MG1655, complete genome.
gggtgcttgaggctgtctgtctcaggcattagctgaacggcagatagaga
aaagccccgagtgatattttaccatcaacccgaggcctcctatatgctga
acacatgtagagtgcctcttactgaccgtaaggtcaaggagaagagagca
atg

Number of predicted targets: 10
Rank Target Energy p-value Probability
1 yghJ (b4466) -11.49 1.1e-06 0.66
2 ypjA (b2647) -12.34 2.6e-06 0.64
3 ivbL (b3672) -23.72 1.0e-04 0.56
4 cadC (b4133) -18.44 2.5e-04 0.54
5 ompF (b0929) -11.26 3.3e-04 0.54

U00096 CMSEARCH misc_RNA 2025227 2025313 102.8 - . UID=misc_RNA.10097;E-value=3.4e-20;Name=DsrA;
Expected interactions with RpoS, possibly hns, rbsD, argR, ilvI

>U00096; Escherichia coli str. K-12 substr. MG1655, complete genome.
aacacatcagatttcctggtgtaacgaattttttaagtgcttcttgctta
agcaagtttcatcccgaccccctcagggtcgggattt

Number of predicted targets: 8
Rank Target Energy p-value Probability
1 yghJ (b4466) -10.49 1.9e-07 0.64
2 mraZ (b0081) -17.75 1.5e-05 0.57
3 ompF (b0929) -12.21 5.0e-05 0.55
4 ypjA (b2647) -7.82 1.1e-04 0.53
5 oppA (b1243) -11.54 1.4e-04 0.52

U00096 CMSEARCH misc_RNA 507204 507287 83.0 + . UID=misc_RNA.10155;E-value=3.1e-12;Name=sroB;
Expected interactions with YbfM, DpiA/DpiB, chbBC intergenic region.
>U00096; Escherichia coli str. K-12 substr. MG1655, complete genome.
acaccgtcgcttaaagtgacggcataataataaaaaaatgaaattcctct
ttgacgggccaatagcgatattggccattttttt

Number of predicted targets: 6
Rank Target Energy p-value Probability
1 ybaK (b0481) -99.5 3.8e-09 0.72
2 yghJ (b4466) -11.05 1.6e-07 0.66
3 ypjA (b2647) -11.82 4.4e-07 0.64
4 ykiD (b4732) -17.31 6.8e-05 0.55
5 mraZ (b0081) -11.74 1.7e-04 0.53

OxyS RNA

Expected interactions with oxyR, yobF, ybaY

>U00096.3:c4158394-4158285 Escherichia coli str. K-12 substr. MG1655, complete genome
GAAACGGAGCGGCACCTCTTTTAACCCTTGAAGTCACTGCCCGTTTCGAGAGTTTCTCAACTCGAATAAC
TAAAGCCAACGTGAACTTTTGCGGATCTCCAGGATCCGCT

Number of predicted targets: 10
Rank Target Energy p-value Probability
1 oxyR (b3961) -151.82 5.9e-09 0.72
2 yghJ (b4466) -8.64 1.0e-06 0.63
3 mreB (b3251) -10.42 1.6e-06 0.63
4 ypjA (b2647) -8.73 4.6e-06 0.61
5 eutJ (b2454) -8.11 1.3e-05 0.59

**Second round of review**

**Reviewer 1**

The author has addressed all my previous comments. A minor suggestion is to include the Genome Accession number in Supplementary Table 1.

**Reviewer 2**

I'm satisfied with the responses to review. Many thanks. Great job!
